# Revisiting the historical scenario of a disease dissemination using genetic data and Approximate Bayesian Computation methodology: The case of *Pseudocercospora fijiensis* invasion in Africa

**DOI:** 10.1002/ece3.10013

**Published:** 2023-04-19

**Authors:** A. Gilabert, A. Rieux, S. Robert, R. Vitalis, M.‐F. Zapater, C. Abadie, J. Carlier, V. Ravigné

**Affiliations:** ^1^ Université de la Réunion, UMR PVBMT Saint‐Pierre France; ^2^ CIRAD, UMR PHIM Montpellier France; ^3^ PHIM Plant Health Institute Univ Montpellier, CIRAD, INRAE, Institut Agro, IRD Montpellier France; ^4^ CIRAD, UMR PVBMT Saint‐Pierre France; ^5^ CBGP Univ Montpellier, CIRAD, INRAE, Institut Agro, IRD Montpellier France; ^6^ Present address: CIRAD, UMR AGAP Institut Montpellier France; ^7^ Present address: UMR AGAP Institut, Univ Montpellier, CIRAD, INRAE, Institut Agro Montpellier France

**Keywords:** Approximate Bayesian Computation, emerging disease, population genetics, random forest

## Abstract

The reconstruction of geographic and demographic scenarios of dissemination for invasive pathogens of crops is a key step toward improving the management of emerging infectious diseases. Nowadays, the reconstruction of biological invasions typically uses the information of both genetic and historical information to test for different hypotheses of colonization. The Approximate Bayesian Computation framework and its recent Random Forest development (ABC‐RF) have been successfully used in evolutionary biology to decipher multiple histories of biological invasions. Yet, for some organisms, typically plant pathogens, historical data may not be reliable notably because of the difficulty to identify the organism and the delay between the introduction and the first mention. We investigated the history of the invasion of Africa by the fungal pathogen of banana *Pseudocercospora fijiensis*, by testing the historical hypothesis against other plausible hypotheses. We analyzed the genetic structure of eight populations from six eastern and western African countries, using 20 microsatellite markers and tested competing scenarios of population foundation using the ABC‐RF methodology. We do find evidence for an invasion front consistent with the historical hypothesis, but also for the existence of another front never mentioned in historical records. We question the historical introduction point of the disease on the continent. Crucially, our results illustrate that even if ABC‐RF inferences may sometimes fail to infer a single, well‐supported scenario of invasion, they can be helpful in rejecting unlikely scenarios, which can prove much useful to shed light on disease dissemination routes.

## Graphical abstract

In this study, we illustrate that, in some cases, accepting not to use available historical data can be a fruitful way of reconsidering invasion processes using the model of the worldwide emergence of the fungal banana pathogen *Pseudocercospora finjiensis*. Using an ABC‐RF approach, we investigated the history of the invasion of Africa by *P. fijiensis* by testing the historical hypothesis against other plausible hypotheses. We do find evidence for an invasion front consistent with the historical hypothesis, but also for the existence of another front never mentioned in historical records and question the historical introduction point of the disease on the continent.

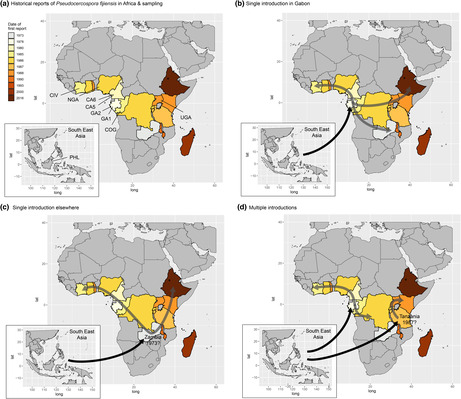

## 1 INTRODUCTION

Over the last two decades, the reconstruction of invasion routes has completely modified the common understanding of invasion processes. The textbook invasion, described as a propagule being introduced from a native area to an invaded area, is now considered as an exception. Multiplicity of native populations, multiple introductions, and admixture, as well as bridgehead populations (i.e., invasive populations that subsequently serve as a source of additional introductions), have almost systematically been detected (Bertelsmeier et al., 2018; Garnas et al., 2016; Gladieux et al., 2015; Guillemaud et al., 2011). This is particularly the case for agricultural pests and opportunist species inhabiting perturbed environments, for which the uniformity of the environment at large spatial scales facilitates regional and global invasions (Hufbauer et al., 2012).

Reconstructing invasion routes at large spatial scales can seldom be conducted on the basis of demographic surveys only (but see Hovmøller et al., 2008) due to the stochastic nature of long‐distance dispersal events. The field has therefore much benefitted from the use of population genetics (Cristescu, 2015), and more specifically the development of inference methods enabling the confrontation of colonization scenarios to genetic data (Estoup & Guillemaud, 2010). Among these methods, Approximate Bayesian Computation (ABC) has played a central role by allowing the exploration of unprecedentedly complex demographic scenarios (Bertorelle et al., 2010; Csilléry et al., 2010). Indeed, contrary to other statistical methods such as maximum likelihood methods, ABC does not rely on the computation of the likelihood, which can be tedious or even impossible to compute for complex demographic models. In ABC, the likelihood is approximated by simulating data sets under considered scenarios (typically using the coalescent framework) and selecting the simulated data sets that are closest to the observed data using a regression approach based on summary statistics (Beaumont et al., 2002). Since their emergence, ABC methods have been continuously improved (Lintusaari et al., 2017; Marin et al., 2012; Sunnåker et al., 2013). Recently, a novel ABC approach (ABC‐RF) based on random forests, a machine learning tool, has been developed in the context of model selection and inference of demographic parameters (Collin et al., 2021; Pudlo et al., 2016; Raynal et al., 2019). Compared to standard ABC methods, the ABC‐RF method is less computationally‐demanding as it requires fewer data sets to efficiently discriminate between scenarios and estimate the posterior probability of the best one. Furthermore, it does not rely on a tolerance level and is more robust to the choice of the summary statistics. This approach has recently been successively used to distinguish between complex demographic scenarios (e.g. Estoup et al., 2018; Fraimout et al., 2017; Javal et al., 2019; van Boheemen et al., 2017) and to estimate demographic parameters (Chapuis et al., 2020; Collin et al., 2021).

ABC methods allow to combine both genetic and historical data. In a few studies, it has been formally proven that combining historical and genetic data enhances the power of ABC approaches (Estoup et al., 2010; Milgroom & Peever, 2003). Typically, historical data can be used to refine and restrain the set of scenarios under consideration (Estoup & Guillemaud, 2010; Fraimout et al., 2017; Lombaert et al., 2014). Historical data can also be used as prior information, allowing to limit the range of prior distributions of parameters such as the dates of introduction events (Estoup et al., 2010; Lombaert et al., 2010). Although the idea seems intuitive that combining historical and genetic data is always better than using genetic or historical data alone, it relies on the assumption that, even when rare, historical data are ‐to some extent‐ reliable. There are undoubtedly biological systems where invasive species are easy to identify and/or under extensive survey, and for which historical data will represent the timing of introduction events rather faithfully. But there is also a myriad of species, among which many plant pathogens, for which identification requires expert skills or laboratory tools, and which might therefore remain undetected for long periods after their introduction into a new environment. Also, upon introduction, many species do pass through a lag phase where population sizes may stay low for some time before important demographic growth. In addition, in the domain of agriculture, the first mention of a crop pathogen may be subject to political censure due to the potentially large consequences of quarantine rules that would follow such official notification. Therefore, depending on species, ecosystems, and countries, historical data may be imprecise or biased.

For such situations, ABC inference methods can also be used on genetic data only using flexible scenarios and uninformative priors, hopefully providing both a way to reconstruct the routes of invasions and to statistically test the reliability of historical data. There have been a number of studies where invasion routes have been reconstructed mainly using genetic data. Genetic data have often confirmed, and even refined the invasion histories reconstructed by the means of historical, geographical, and ecological data (e.g., Croucher et al., 2013), while sometimes revealing discrepancies (Barun et al., 2015; Cristescu, 2015; Hoos et al., 2010).

**FIGURE 1:**
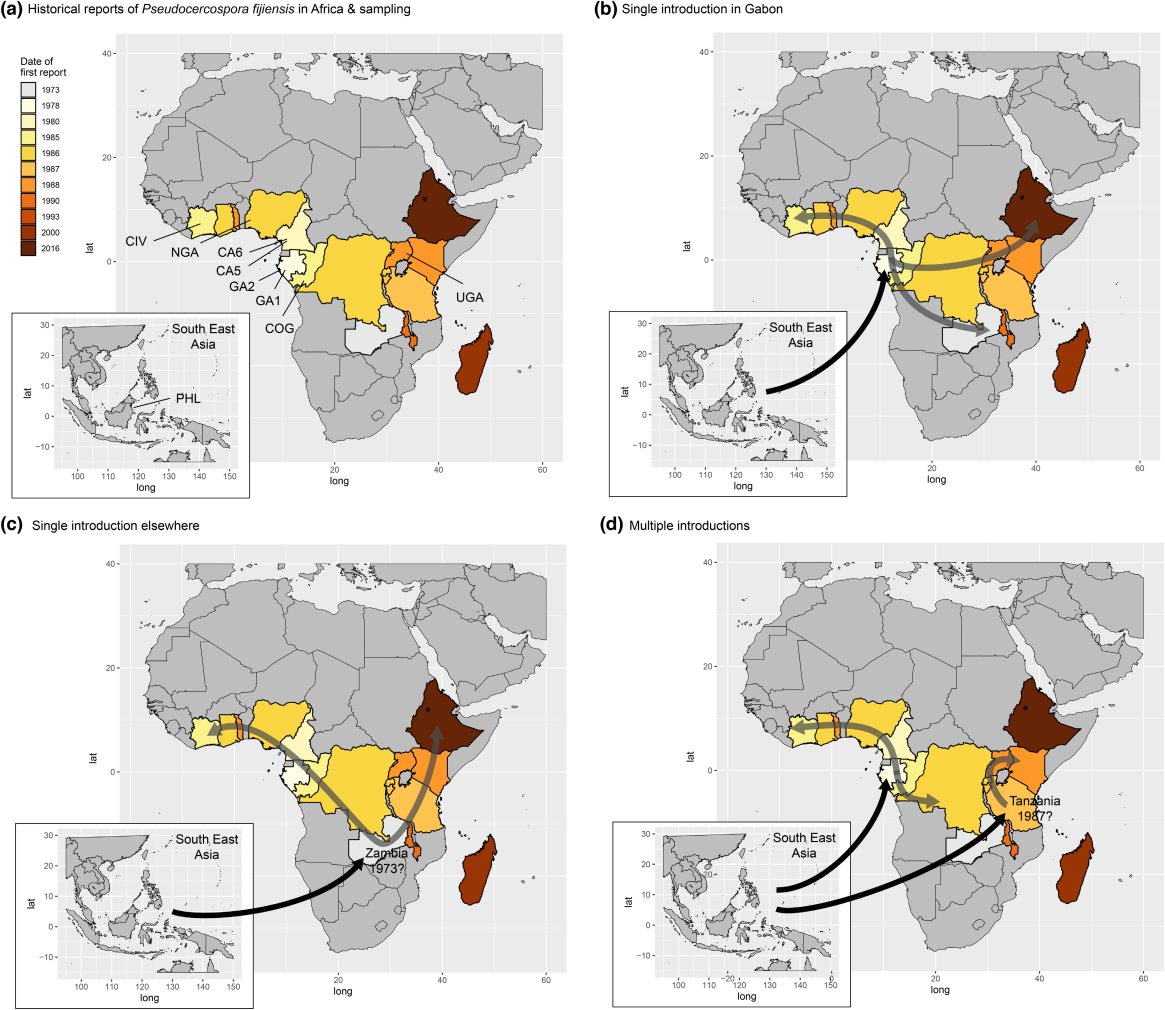
The invasion of the African continent by *Pseudocercospora fijiensis*. (a) First reports of black leaf streak disease in Africa, adapted from Blomme et al. (2013), and populations sampled. The countries are colored according to the date of first report of the disease, the darker the more recent report. (b) Historical data mention the introduction into Gabon of infected plant material brought from Asia, in 1978, leading to the main hypothesis of an invasion of Africa from Gabon. (c) A potential observation of the disease reported in Zambia in 1973 suggests that a more central introduction might have occurred before that into Gabon, but this observation was never officially validated. Gabon might therefore not be the introduction point. (d) The origin of Central and East African contaminations remains unknown, but a later independent introduction into a coastal area of East Africa in 1987 (Pemba Island, Tanzania) might have led to the spread of the disease along the East African coast and toward the interior.

Eventually, ignoring historical data to constrain the set of possible scenarios explored will often come at a cost in terms of statistical power. The worldwide emergence of the fungal banana pathogen *Pseudocercospora fijiensis*, which causes black leaf streak disease (BLSD), provides a suitable model to address this issue. BLSD is the most destructive banana leaf disease and is considered as among the most serious threats to global food security (Pennisi, 2010). Historical data concerning its spread throughout most banana‐growing regions are largely available (Blomme et al., 2013; Guzman et al., 2018). BLSD was first recognized in Fiji in 1963 and then rapidly reported throughout the Pacific, suggesting that it was probably present in the area before its discovery (Jones, 2018). The first report in Africa was in 1973 in Zambia, although there has not been any confirmation that the disease was actually the black leaf streak (Blomme et al., 2013). Since then, no report of BLSD in Zambia has been made (Blomme et al., 2013). The next reports in Africa were in Gabon and São Tomé where symptoms of BLSD were observed (Frossard, 1980 in Blomme et al., 2013). Then, during the 1980s, the disease was observed along the west coast of Africa, in Cameroon, Nigeria, Togo, Ghana, and Ivory Coast (see Figure 1a, Blomme et al., 2013 and references herein). The disease, probably coming from Gabon, was reported in Congo Brazzaville in 1985, and from there may have spread eastward through the Democratic Republic of Congo, Rwanda, Burundi, and Uganda (see Figure 1a, Blomme et al., 2013 and references herein). Another introduction in East Africa may have occurred from Pemba Island where the BLSD has been reported in 1987 (Dabek & Waller, 1990), and from there to Tanzania, Kenya, and Malawi (see Figure 1a, Blomme et al., 2013 and references herein) the last country invaded in Africa in the 1990s. More recently (in 2016), the disease has been detected in Ethiopia (Gurmu et al., 2017). Population genetics studies have revealed that, despite its wind‐dispersed ascospores and its widespread sexual reproduction, *P. fijiensis* is one of the most genetically structured fungal crop pathogens, even at country‐wide or continental scales (Rivas et al., 2004; Robert et al., 2012). The study of a worldwide sample of *P. fijiensis* populations using a combination of clustering analyses based on microsatellite markers and phylogenetic network approaches applied to sequence markers suggested that the introduction of a small number of genotypes from a single South‐East Asian source population led to invasion of the whole African continent (Robert et al., 2012). The high levels of genetic differentiation reported in Africa further suggested that range expansion was stochastic, with frequent bottlenecks accompanying population establishment (Rivas et al., 2004; Robert et al., 2012). This scenario challenges the historical hypothesis with at least two independent introductions: (i) one introduction near Gabon (Blomme et al., 2013, Figure 1a), followed by two gradual geographical propagation fronts, in the whole of western Africa (Mourichon & Fullerton, 1990) and in East Africa, and (ii) one introduction in East Africa, in an island of the Zanzibar Archipelago (Blomme et al., 2013). These discrepancies between historical data and population genetic structure are such that the scenarios of introduction and subsequent intra‐continental spread in Africa remain poorly understood. The reliability of historical data could be questioned here, as *P. fijiensis* causes symptoms easily mistaken for those of another fungal pathogen also present in most African countries, *Pseudocercospora musae*. In this paper, we applied recent ABC methodological developments to African populations of *P. fijiensis*, and used the increased precision of these methodologies to put historical data at a test in the case of a devastating plant pathogen.

We aimed to test the validity of these historical hypotheses of introduction in Gabon followed by a continuous propagation to the whole of West Africa, and possibly East Africa. To that end, we analyzed the genetic structure of eight pathogen populations from six eastern and western African countries, using 20 microsatellite markers and carried out an ABC‐RF model choice analysis to reconstruct the history of the invasion of Africa by *P. fijiensis*. We illustrate that even in situations of limited discriminative power, focussing on hypothesis rejection may shed new lights on the historical expansion of diseases at large spatial scales.

## 2 MATERIALS AND METHODS

### 2.1 Sampling and genotyping

The samples were obtained from the historical collection from the CIRAD stored in glycerol at −80°C (see Robert et al., 2012 for more details on the sampling collection). Eight populations from six countries in Africa (Uganda, Congo, Gabon, Cameroon, Nigeria, and Ivory Coast) and one population from South‐East Asia collected from around the South China Sea (the Philippines) were sampled at different times (Table 1). All populations consisted of individuals isolated during the same time period from different plants from the same banana field. The host plants were susceptible banana clones from the AAA and AAB genome groups, as defined by their genomic composition (i.e., triploid cultivars with three sets of chromosomes from *Musa acuminata* for the AAA group or with two sets of chromosomes from *Musa acuminata* and one set from *Musa balbisiana*). Each *P. fijiensis* individual corresponded to a fungal culture strain derived from a single ascospore (i.e., sexually produced spore) isolated from necrotic infectious banana leaf tissue as described in Zapater et al. (2008). All populations except for the population from Congo (COG) had previously been genotyped using 21 microsatellites in a global‐scale study of *P. fijiensis* history (Robert et al., 2012). Strain culture, DNA extraction, and microsatellite genotyping with the 21 microsatellite markers were carried out for the population COG as described in Robert et al. (2012). One microsatellite marker (MfSSR355) was monomorphic at the scale of Africa and was discarded; all the analyses in this study were therefore performed using 20 markers. The microsatellite dataset are deposited in the dryad repository: doi:10.5061/dryad.rn8pk0pgw.

**TABLE 1:** Sampling data and genetic diversity of the nine *Pseudocercospora fijiensis* populations collected in six countries in Africa and in Philippines.

Geographic area	Country	Population	Sampling date		Sample size	*H*_*s*_	*A*_*r*_	*pA*_*r*_
			Year	nb of generations since the last sampling				
AFRICA	Uganda	UGA	1998	130	34	0.28	1.9	0.08
	Cameroon	CA5	2001	100	21	0.21	1.6	0.06
		CA6	2001	100	24	0.17	1.6	0.01
	Ivory Coast	CIV	1999	120	34	0.14	1.5	0.01
	Gabon	GA1	1998	130	10	0.07	1.1	0
		GA2	1998	130	10	0.19	1.5	0.06
	Congo	COG	2011	0	32	0.18	1.7	0.17
	Nigeria	NGA	1999	120	24	0.1	1.4	0
	Average	‐	‐		‐	0.17	1.5	‐
S‐E ASIA	Philippines	PHL	1993	180	23	0.6	4.2	2.69

### 2.2 Genetic diversity and population differentiation

Population and diversity indices were estimated from a diploidised dataset, after re‐coding haploid genotypes into diploid ones. Gametic linkage equilibrium between all pairs of loci was tested in all populations, by Fisher's exact tests implemented in genepop_4.0_ (Raymond & Rousset, 1995; Rousset, 2008). We used the false discovery rate (FDR) procedure implemented in the R package qvalue (Storey, 2002) to control for multiple testing. Gene diversity *H*_*s*_ was estimated following Nei (1987) with fstat_2.9.3_ (Goudet, 1995). We used the rarefaction method implemented in hp‐rare (Kalinowski, 2005) to calculate the allelic richness *A*_*r*_ and private allelic richness *pA*_*r*_, for standardized sample sizes of 20 individuals per population. Two different indices were used for estimating genetic differentiation between African populations: the pairwise *F*_ST_ (Weir & Cockerham, 1984), which are affected by migration rates and effective population sizes and were calculated using fstat_2.9.3_ (Goudet, 1995), and the pairwise Jost *D* (Jost, 2008), which strictly reflect the genetic distance between populations and were computed using the program Smogd web version 1.2.5 (Crawford, 2010). Jost *D* are based on the effective number of alleles and measure the relative degree of allelic differentiation between populations and are supposed to be less constrained by the level of polymorphism of the markers than the pairwise *F*_ST_ (see Jost, 2008 for further information on Jost *D*). The genetic relationships between African populations, based on the Cavalli‐Sforza chord distance, were represented graphically using an unrooted neighbor‐joining (NJ) tree as implemented in populations‐1.2.32 (http://bioinformatics.org/~tryphon/populations/#ancre_telechargement). Node supports were obtained by implementing 1000 bootstraps. Tree visualization was done using the R packages treeio (Wang et al., 2020) and ggtree (Yu et al., 2017).

It should be noted that the low number of Gabonese isolates may bias the results, with an underestimation of genetic diversity within the Gabonese populations and an overestimation of population differentiation between any population pair that includes a Gabonese population. We thus tested if genetic diversity (allelic richness *A*_*r*_ and genetic diversity *H*_*s*_) within the Gabonese samples was significantly lower than in the other samples using the test of comparison among groups of samples implemented in fstat_2.9.3_ (Goudet, 1995) with 1000 permutations, and whether population differentiation (estimated by the means of the pairwise *F*_ST_) was significantly greater when a Gabonese population was concerned using a Wilcoxon test implemented in R v.4.1.0 (R Core Team, 2021).

### 2.3 Clustering analyses

Spatial genetic structure in African samples was investigated with the model‐based procedure implemented in structure (Pritchard et al., 2000), to assign individual genotypes to a predetermined number of clusters *K*, assuming linkage equilibrium between loci within clusters. We conducted a series of 10 independent runs for each value of *K*, with *K* varying from 1 to 10, using the admixture model. Each run consisted of 10^6^ iterations after a burn‐in period of 3 x 10^4^ steps. Markov chain‐Monte Carlo (MCMC) convergence and consistency between the 10 replicate runs were examined for each value of *K*. The optimal number of clusters was determined using the Delta(*K*) method (Evanno et al., 2005) based on the second‐order rate of change of Ln[Pr(*D*|*K*)], implemented in structure harvester web version 0.6.94 (Earl & vonHoldt, 2012). Clustering results of independent replicate runs were checked for congruence and optimally aligned using Clumpp version 1.1.2 (Jakobsson & Rosenberg, 2007) and final results were represented with distruct version 1.1 (Rosenberg, 2004). To corroborate those results using a different clustering approach, we also performed a Discriminant Analysis of Principal Components analysis (DAPC, Jombart et al., 2010) implemented in the adegenet package (Jombart, 2008; Jombart & Ahmed, 2011). Because the population COG from Congo has been sampled 10 years after the other African populations, which can introduce bias in the analysis, we performed the two analyses, one including all the African populations and one without the population from Congo.

### 2.4 ABC inference of the pathways of dissemination across Africa

ABC scenario choice was carried out, to compare the historical hypothesis of a single western Gabonese introduction (Figure 1b) with a hypothesis of several introductions (as suggested by the high *F*_ST_ values between countries, see Results; Figure 1d), and with that of a single introduction elsewhere (Figure 1c). To do so, we used the ABC methodology relying on the Random Forest statistical tool, named ABC‐RF and implemented in the R package abcrf (Pudlo et al., 2016).

#### 2.4.1 Demographic scenarios

**FIGURE 2:**
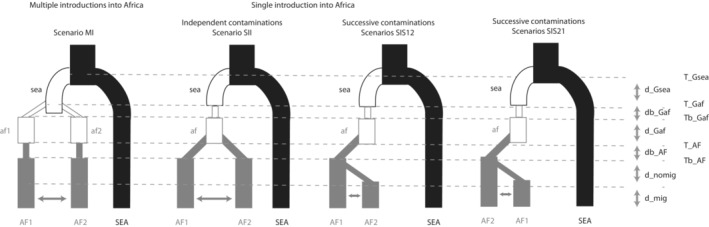
Scenarios compared in ABC‐RF analysis. Scenario MI (Multiple Introductions) corresponds to the scenarios with two independent introductions into Africa from the same Asian origin while scenarios SI (Single Introduction) corresponds to the scenarios where Africa was invaded through a single introduction event from Asia. Scenario SII (Single introduction, Independent contaminations) represents the scenario where, after a single introduction from Asia, the African continent has been invaded following two independent contaminations. Scenarios SIS12 and SIS21 (Single introduction, Successive contaminations) correspond to the scenarios where after a single introduction into Africa, the continent was then colonized through successive contaminations. SEA is a sampled South East Asian population, sea an unsampled South East Asian population, af_*i*_ the unsampled African populations, and AF_*i*_ the sampled African populations. Thin lines reflect effective population size reductions (bottlenecks). Solid thick lines represent sampled populations and hollow lines represent unsampled populations. Black lines correspond to ancestral South East Asian population divergences and gray lines correspond to events occurring within Africa. We used a ‘*d*’ as a prefix to name parameters of length of time: d_mig/d_nomig, the length of time with/without migration between AF1 and AF2, d_Gsea/dGaf, the period of time of the established unsampled (Ghost) sea and af populations (i.e., for the af populations, after the reduction of population size that followed the introduction). Bottleneck durations are expressed using the ‘db’ prefix. Parameters with names starting with a ‘*T*' are the timing of events, the origin of populations (T_Gsea, T_Gaf and T_AF) and the end of the bottleneck period (Tb_Gaf and Tb_AF). For the definition and priors, see Appendix S1

The final demographic scenario of the invasion of Africa by *P. fijiensis* was reconstructed by combining the results of simple three‐population‐based ABC‐RF analyses. We ensured that all putative scenarios were covered in an exhaustive manner, in a tractable analysis, by restricting the analysis to three sampled populations at a time (Figure 2): the South‐East Asian sample (hereafter denoted SEA) and two African samples (denoted AF1 and AF2). Previous analyses strongly suggested that none of the SEA populations sampled were the initial source of the African introduction (Figure 5 in Robert et al., 2012). We therefore considered that the African populations diverged from an unsampled Asian population (hereafter denoted sea and represented with a hollow black line in Figure 2), rather than the sampled SEA populations. Similarly, we considered that we did not sample the African populations at the origin of the invasion but that the African populations we sampled diverged from unsampled African populations (denoted af1 and af2 and represented with hollow gray lines in Figure 2).

The possibility of multiple introductions from two different Asian sources was not considered given the very low diversity in Africa on both microsatellite and sequence data (Robert et al., 2012). Only separate introductions from the same Asian source were tested using a scenario that considered the African populations AF1 and AF2 to descend from two unsampled African founder populations, af1 and af2, originating themselves from the unsampled Asian population sea (scenario MI, Figure 2). This scenario was compared to others with a single introduction into Africa (SI scenarios). Among these, we designed scenarios to test whether AF1 and AF2 derived from independent contaminations from an unsampled African founder population af (SII, Figure 2) or whether one of these populations descended from the other (SIS12 & SIS21 scenarios, Figure 2). Because migration might have occurred after the establishment of the actual African populations, for each one of these scenarios we considered a scenario allowing migration (MI_mig, SII_mig, SIS12_mig & SIS21_mig) and another one without migration (MI_nomig, SII_nomig, SIS12_nomig & SIS21_nomig) between the two African sampled populations AF1 and AF2 (see Figure 2). We then compared the eight scenarios systematically, using ABC‐RF scenario choice for all possible different pairs of African samples (i.e., 28 pairs of African populations). Those model choice analyses allowed us to accept or to rule out some scenarios to describe the relationships between pairs of African populations to reconstruct a final scenario including most sampled African.

#### 2.4.2 Prior distributions of the parameters

Appendix S1 provides the prior distributions of model parameters, including population sizes, mutation rates, and divergence times. Time was expressed in numbers of generations, assuming 10 successive generations per year (Churchill, 2011). Within Africa, we modeled effective size variations at each population foundation.

#### 2.4.3 Model choice analyses

We simulated 40,000 datasets for each of the eight scenarios using fastsimcoal v2.6 (Excoffier et al., 2013; Excoffier & Foll, 2011). An example of the template and estimation files required for fastsimcoal is given for each scenario in the Dryad repository (repository available upon acceptance). ABC methods involve the comparison of observed and simulated datasets on the basis of summary statistics. In this study, we computed the following summary statistics using arlsumstat v.3.5.2, a command line version of arlequin (Excoffier & Lischer, 2010): the mean and standard deviation over loci of population‐specific number of alleles, allelic range, heterozygosity, and Garza‐Williamson's *M* index (Excoffier et al., 2005) which was computed as in Garza and Williamson (2001) and also in a modified version to deal with monomorphic populations; we further used the mean and standard deviation over loci of averages over populations of the number of alleles, the allelic range, the heterozygosity and the *M* index. We evaluated the quality of the simulated models (scenarios and prior definitions) using Principal Components Analyses (PCAs) to project the summary statistics obtained from simulated and observed data and by investigating for each summary statistic whether the observed value falls within the simulated values drawn from prior distributions. PCAs were performed and visualized using the R packages abc v.2.1 Csilléry et al., 2012. For each summary statistic we plotted its distribution using the R package ggplot2 v3.3.5 (Wickham et al., 2016) and calculated the number of simulations for which we obtained a summary statistic higher than the observed one. Then, we used the R package abcrf v1.7.1 (Pudlo et al., 2016) to provide a classification vote for the eight scenarios representing the number of times a scenario is selected as the model best fitting the dataset among a forest of 3000 trees, and in a second step the posterior probability of the best‐selected model. The number of simulated datasets per scenario and the number of trees in the random forests were chosen to ensure the stability of the prior error rate estimates. We evaluated the influence of both the size of the reference table and the number of trees in the RF being evaluated for three trios of populations (Appendix S2) and used the same values for the 25 analyses. ABC‐RF model choice analyses quality and consistency were evaluated by calculating the prior error rate and by replicating the analyses 10 times.

## 3 RESULTS

### 3.1 Genetic diversity and population differentiation

None of the pairs of loci presented significant linkage disequilibrium after FDR control. Average genetic diversity was much lower in Africa (*H*_s_: 0.17, *A*_r_: 1.6) than in South‐East Asia (*H*_e_: 0.6, *A*_r_: 3.1), with very few private alleles. UGA was the most diverse African population (*H*_s_: 0.28; *A*_r_: 1.9; *pA*_r_: 0.07). Private allele richness was highest in the COG population (*pA*_r_: 0.15), which might stem from COG being sampled about 100 generations after the other populations (Table 1). It is worth mentioning that one of the populations from Gabon, GA1, did not have any private allelic richness. Apart from the pair GA2/CA5, which *F*_ST_ value was null, African populations were highly differentiated with pairwise *F*_ST_ values ranging between 0.13 and 0.55 (Appendix S3a), whatever the geographic distance between them. By contrast, Jost D values were all below 0.06 (data not shown), suggesting that they might be poor indicators of differentiation in our case, as they could have been strongly impacted by the fact that African populations would be out of mutation‐drift equilibrium (Ryman & Leimar, 2009). Despite the lower number of isolates in the Gabonese populations, genetic diversity (both allelic richness *A*_*r*_ and genetic diversity *H*_*s*_) did not appear to be significantly lower in these samples (*p*‐values *A*_*r*_ = .07, *H*_s_ = .21). Similarly, pairwise *F*_ST_ did not appear to be significantly higher when the population pair included a Gabonese population (Wilcoxon test, W = 142, *p‐*value = .32).

A graphical representation of the genetic relationships between the populations included in this study is given in Appendix S3b, which shows the unrooted NJ reconstructed based on the Cavalli‐Sforza chord distance. This tree shows a closer relationship between the population PHL, the population from the Philippines representing the population source of the African populations from south‐east Asia, with the populations from Congo (COG) and from Uganda (UGA), and a relative proximity between the populations GA1 from Gabon and NGA from Nigeria compared to the others.

### 3.2 Clustering analyses

**FIGURE 3:**
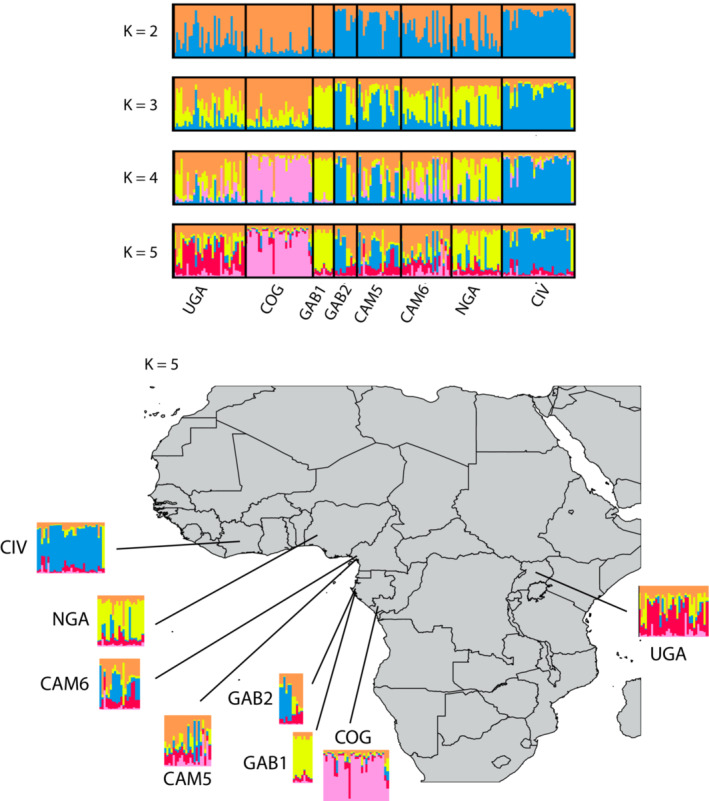
Bayesian clustering of African multilocus microsatellite haplotypes, using structure for *K* = 2 to *K* = 5. Each individual is represented by a vertical line, divided into up to *K*‐colored segments representing the individual's estimated likelihood of membership of each of the *K* clusters. Vertical black lines separate individuals from the different populations of origin, as indicated by population abbreviations under the bar plot (detailed in Table 1)

Applying Evanno's Δ*K* to the output of STRUCTURE analysis pointed *K* = 5 as the best model when all the African populations were included (Appendix S4). When increasing *K* to this value, the most divergent groups would be expected to separate into distinct clusters first (Pritchard et al., 2000). Here, a mixture of clusters was observed within all countries, regardless of the value of *K* considered (Figure 3). However, for *K* = 5, COG and CIV were clearly separated from the other populations because, for each of these, all individuals were preferentially assigned to one cluster (pink for COG, blue for CIV). Individuals from NGA and GA1 were mainly assigned to the same cluster (yellow). A dominance of the red cluster was observed in UGA, while individuals from GA2, CA5, and CA6 had totally mixed ancestries in multiple clusters. The DAPC analysis did not allow to clearly identify a number of clusters, but from *K* = 4, increasing the number of clusters did not improve significantly the fit (i.e., the BIC values decreased marginally), and a similar structuration to that identified using STRUCTURE was observed for *K* = 4 or 5: the populations COG and CIV were differentiated from the others, which were distributed between the other clusters without a clear separation among them (results not shown).

When we removed the more recently sampled population (COG population), the best model identified using the Evanno's Δ*K* was for *K* = 3 (Appendix S5). Considering three genetic clusters, we observed a similar structuration when considering the COG population or not (Appendix S5). The population CIV, GA1, and NGA were relatively homogeneous with most of the individuals from CIV being preferentially assigned to the blue cluster and most of the individuals from GA1 and NGA being preferentially assigned to the yellow cluster. The individuals from the other populations showed mixed ancestries in the three clusters with different proportions.

### 3.3 ABC scenario choice: general trends

Graphic representations of the summary statistics (PCA and their distribution) revealed that the simulations performed under the eight scenarios using the parameter values drawn from prior distributions were relatively close to the observed dataset. First, the representation of the PCA in the space of the summary statistics showed that the observed dataset fell well within the simulated ones (Appendix S6). Second, most of the observed summary statistics did not appear to deviate from the distributions of the summary statistics computed from the simulated dataset (see Appendix S7 as an illustration using the trio of populations PHL, CIV, and CA6). Three summary statistics were consistently found to be at the right extremity of the distribution: the standard deviation over loci of the number of alleles and of heterozygosity in the PHL population (Ksd_3 and Hsd_3) and the standard deviation over loci of the Garza‐Williamson's *M* index of some African populations (GWsd_2). Three other summary statistics were often found at the right extremity of the distribution: the standard deviation over loci and populations of the heterozygosity (sd_H) and of the Garza‐Williamson's *M* index (sd_NGW), and the standard deviation over loci of some African populations (GWsd_1; see Appendix S8). In addition, we plotted the variable importance plot for the 10 replicates for two population triplets, one triplet with consistent results over the 10 replicates and one scenario showing a relatively clear higher percentage of votes compared to the other scenarios, and one with inconsistent results over the replicates, with multiple scenarios showing a relatively similar percentage of votes (Appendix S9). For each population triplets, the first 10 most important variables were, to a few exceptions, the same for the 10 replicates and did not include the statistics that were found to be outside the distribution. Those observations suggest that those later statistics were not responsible for the classification results.

Table 2 summarizes the results of the ABC‐RF model choice analyses while detailed results of all ABC‐RF tests are presented in Appendix S10 and commented below. Over all ABC scenario choice tests, many provided moderate posterior probabilities, and/or conflicting percentages of votes among replicate tests. The following results are thus based on (i) the rejection of scenarios with low percentage of votes and (ii) the interpretation of scenarios with consistent votes among the 10 replicates of the test.

**TABLE 2:** Results of the ABC‐RF model choice analyses.

		Ca5 & civ	Ca6 & civ	Civ & Ga1	Civ & Ga2	Civ & Nga	Cog & Ca5	Cog & Ca6
Selected scenario		SIS12	SIS_mig	**SIS12_nomig**	SIS21	SIS12_mig	mig	mig
		6 nomig 4 mig	9 mig		6 mig 4 nomig	9 mig 1 nomig	mig	mig
		SIS12	8 SIS12 2 SIS21		9 SIS21 1 SIS12	9 SIS12 1 SIS21	8 SIS12 2 SIS21	SII / SIS
Prior error rate (SD)				**0.407 (0.022)**				
Posterior probability (SD)				**0.723 (0.001)**				
Scenarios % votes (SD)	MI_mig	6.7% (0.012)	7.0% (0.014)	**3.9% (0.008)**	5.8% (0.012)	6.1% (0.015)	8.5% (0.011)	9.9% (0.012)
	MI_nomig	6.8% (0.014)	5.5% (0.013)	**12.4% (0.014)**	5.9% (0.008)	4.1% (0.005)	6.0% (0.017)	4.8% (0.006)
	SII_mig	11.5% (0.024)	11.7% (0.011)	**8.0% (0.020)**	11.2% (0.016)	11.6% (0.016)	14.8% (0.022)	17.0% (0.011)
	SII_nomig	10.1% (0.015)	9.5% (0.010)	**16.3% (0.015)**	10.3% (0.010)	8.6% (0.014)	9.3% (0.007)	8.6% (0.010)
	SIS12_mig	19.7% (0.025)	20.9% (0.020)	**14.1% (0.013)**	17.4% (0.017)	21.3% (0.019)	19.9% (0.020)	19.1% (0.022)
	SIS12_nomig	20.6% (0.021)	17.9% (0.024)	**27.9% (0.034)**	10.3% (0.012)	19.3% (0.019)	14.2% (0.015)	14.2% (0.014)
	SIS21_mig	15.2% (0.012)	17.7% (0.025)	**11.5% (0.020)**	19.5% (0.024)	18.3% (0.017)	17.4% (0.017)	17.2% (0.011)
	SIS21_nomig	9.5% (0.015)	9.8% (0.011)	**5.7% (0.005)**	19.6% (0.023)	10.6% (0.016)	9.9% (0.014)	9.3% (0.010)

		Cog & civ	Cog & Ga1	Cog & Ga2	Cog & Nga	Ca5 & Uga	Ca6 & Uga	Civ & Uga
Selected scenario		**SIS12_nomig**	**SIS12_nomig**	mig	**SIS12_nomig**	**SIS21_nomig**	**SIS21_nomig**	**SIS21_nomig**
				mig				
				SII / SIS				
Prior error rate (SD)		**0.404 (0.022)**	**0.450 (0.016)**		**0.415 (0.017)**	**0.440 (0.016)**	**0.401 (0.024)**	**0.453 (0.026)**
Posterior probability (SD)		**0.691 (0.001)**	**0.726 (0.001)**		**0.699 (0.001)**	**0.698 (0.000)**	**0.695 (0.001)**	**0.685 (0.001)**
Scenarios % votes (SD)	MI_mig	**5.3% (0.009)**	**3.1% (0.013)**	9.3% (0.010)	**4.6% (0.005)**	**4.2% (0.008)**	**5.8% (0.007)**	**2.6% (0.004)**
	MI_nomig	**9.9% (0.020)**	**22.0% (0.021)**	5.3% (0.006)	**12.4% (0.026)**	**10.6% (0.013)**	**7.3% (0.006)**	**13.7% (0.033)**
	SII_mig	**9.5% (0.016)**	**4.2% (0.008)**	15.4% (0.016)	**8.1% (0.015)**	**7.1% (0.008)**	**10.5% (0.017)**	**5.3% (0.014)**
	SII_nomig	**14.0% (0.020)**	**20.4% (0.019)**	9.2% (0.008)	**17.7% (0.026)**	**15.0% (0.022)**	**11.6% (0.018)**	**21.7% (0.019)**
	SIS12_mig	**16.6% (0.014)**	**7.6% (0.012)**	17.3% (0.015)	**13.4% (0.017)**	**10.1% (0.015)**	**13.2% (0.019)**	**5.6% (0.011)**
	SIS12_nomig	**23.9% (0.026)**	**34.8% (0.020)**	14.6% (0.018)	**29.2% (0.022)**	**4.6% (0.007)**	**6.5% (0.007)**	**2.8% (0.007)**
	SIS21_mig	**13.2% (0.021)**	**4.8% (0.008)**	17.4% (0.011)	**9.8% (0.017)**	**13.3% (0.013)**	**16.7% (0.015)**	**10.3% (0.015)**
	SIS21_nomig	**7.6% (0.010)**	**3.0% (0.008)**	11.5% (0.014)	**4.8% (0.008)**	**35.1% (0.021)**	**28.3% (0.034)**	**38.0% (0.023)**

		Cog & Uga	Ga1 & Uga	Ga2 & Uga	Nga & Uga	Ca5 & Ca6	Ca5 & Ga1	Ca5 & Ga2
Selected scenario		SIS21	**SIS21_nomig**	**SIS21_nomig**	**SIS21_nomig**	SIS_mig	**SIS12_nomig**	SIS_mig
		8 nomig 2 mig				mig		9 mig 1 nomig
		SIS21				7 SIS12 3 SIS21		5 SIS12 5 SIS21
Prior error rate (SD)			**0.457 (0.025)**	**0.366 (0.017)**	**0.490 (0.024)**		**0.450 (0.024)**	
Posterior probability (SD)			**0.724 (0.001)**	**0.724 (0.001)**	**0.695 (0.001)**		**0.728 (0.001)**	
Scenarios % votes (SD)	MI_mig	7.1% (0.009)	**2.1% (0.006)**	**6.2% (0.007)**	**2.4% (0.004)**	7.4% (0.011)	**2.7% (0.006)**	7.1% (0.013)
	MI_nomig	8.9% (0.021)	**21.8% (0.018)**	**7.2% (0.006)**	**15.8% (0.020)**	4.8% (0.005)	**21.8% (0.035)**	4.7% (0.010)
	SII_mig	12.4% (0.010)	**3.3% (0.007)**	**11.4% (0.021)**	**4.6% (0.014)**	12.9% (0.016)	**4.2% (0.010)**	12.1% (0.012)
	SII_nomig	12.6% (0.023)	**25.7% (0.028)**	**11.0% (0.017)**	**23.5% (0.027)**	9.0% (0.012)	**21.4% (0.018)**	8.3% (0.015)
	SIS12_mig	14.2% (0.019)	**2.2% (0.003)**	**14.6% (0.013)**	**4.0% (0.010)**	19.8% (0.015)	**7.1% (0.010)**	19.2% (0.018)
	SIS12_nomig	8.6% (0.013)	**1.4% (0.003)**	**7.3% (0.010)**	**2.1% (0.006)**	15.2% (0.019)	**36.0% (0.020)**	14.3% (0.025)
	SIS21_mig	16.3% (0.025)	**6.2% (0.011)**	**17.7% (0.014)**	**7.8% (0.008)**	18.3% (0.021)	**4.1% (0.007)**	20.1% (0.019)
	SIS21_nomig	19.9% (0.022)	**37.5% (0.025)**	**24.6% (0.021)**	**39.8% (0.022)**	12.6% (0.016)	**2.6% (0.008)**	14.1% (0.023)

		Ca5 & Nga	Ca6 & Ga1	Ca6 & Ga2	Ca6 & Nga	Ga1 & Ga2	Nga & Ga1	Nga & Ga2
Selected scenario		SIS12_nomig	**SIS12_nomig**	SIS_mig	**SIS12_nomig**	SIS21_nomig	SIS12_nomig	SIS21_nomig
		9 nomig 1 mig		mig		nomig	9 nomig 1 mig	9 nomig 1 mig
		9 SIS12 1 SIS21		7 SIS12 3 SIS21		9 SIS21 1 SII	SIS12	SIS21
Prior error rate (SD)			**0.420 (0.019)**		**0.366 (0.025)**			
Posterior probability (SD)			**0.726 (0.001)**		**0.702 (0.001)**			
Scenarios % votes (SD)	MI_mig	6.5% (0.007)	**3.7% (0.009)**	7.8% (0.023)	**6.4% (0.009)**	3.0% (0.008)	4.9% (0.007)	6.0% (0.010)
	MI_nomig	9.1% (0.020)	**15.9% (0.016)**	4.9% (0.008)	**7.1% (0.019)**	20.7% (0.025)	8.0% (0.019)	7.9% (0.017)
	SII_mig	9.3% (0.010)	**6.9% (0.008)**	14.3% (0.023)	**12.0% (0.015)**	4.6% (0.009)	11.2% (0.020)	11.3% (0.024)
	SII_nomig	12.5% (0.021)	**19.6% (0.030)**	8.3% (0.012)	**10.6% (0.016)**	22.3% (0.030)	11.3% (0.014)	13.6% (0.018)
	SIS12_mig	16.0% (0.016)	**11.0% (0.013)**	19.5% (0.019)	**17.4% (0.015)**	4.7% (0.010)	17.1% (0.020)	12.6% (0.019)
	SIS12_nomig	26.9% (0.031)	**31.2% (0.032)**	13.4% (0.014)	**23.0% (0.036)**	2.9% (0.005)	22.1% (0.020)	8.0% (0.013)
	SIS21_mig	12.2% (0.037)	**7.3% (0.016)**	18.8% (0.024)	**15.4% (0.022)**	8.8% (0.013)	14.7% (0.020)	17.2% (0.028)
	SIS21_nomig	7.6% (0.010)	4.4% (0.010)	13.0% (0.020)	**8.2% (0.013)**	33.0% (0.029)	10.7% (0.022)	23.5% (0.034)
	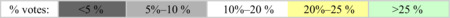							

*Note*: Analyses are identified using the African population pair considered, named as in Table 1. Scenario MI: Multiple Introductions; Scenario SII: Single introduction, Independent contaminations; Scenarios SIS12 and SIS21: Single introduction, Successive contaminations; mig and nomig correspond to scenarios with and without migration between the African populations (see main text and Figure 2 for details). The percentage of votes (for the 28 three‐population tests), prior error rates and posterior probabilities of the best model chosen using ABC‐RF (for the three‐population tests with consistent results over the 10 replicates) were averaged over the 10 replicate analyses. Analyses with consistent results over the replicates are highlighted in bold; for the other three‐population tests, a summary of the different scenarios identified over the 10 replicates is given.

At first glance, our analyses allowed us to reject confidently the scenarios that simulated multiple introductions from the same south‐east Asian source (scenarios MI_mig and MI_nomig), suggesting that all the African populations studied here originated from a single African bridgehead population, which itself originated from a native population from south‐east Asia. Regardless of the African population pairs considered, the MI_mig scenario consistently showed a low to very low percentage of votes (0.01–0.13, mean ± SD = 0.06 ± 0.02; Table 2, Appendices S10 and S11a,b). The situation was less obvious for the scenario without migration, MI_nomig, especially for population triplets that included the population from Gabon GA1. Out of the 28 three‐population tests, 18 showed a mean percentage of votes for this scenario over the 10 replicates below 10% (Table 2, Appendices S10 and S11a,b). For the remaining 10 three‐population tests, although the scenario MI_nomig was not necessarily the less supported scenario and could show up to 26% of votes, other scenarios were always more likely. Among these 10 tests, half included the population GA1 from Gabon, with a relatively high percentage of votes for the scenario MI_nomig.

Out of the 28 three‐population tests, we got consistent results among the 10 replicates for 13 of them (analyses including African populations UGA and either CA5, CA6, CIV, GA1, GA2, or NGA, NGA together with CA6 or COG, GA1 with either CA5, CA6, CIV or COG, and COG with CIV).

### 3.4 ABC scenario choice: two historical fronts go through Western Africa

The ABC‐RF analyses coupled with the results of the private allelic richness and pairwise genetic differentiation support the historical hypothesis of a propagation front starting in Gabon to the West of Africa but also indicate the presence of at least a second invasion front going through Western Africa and originating from Eastern Africa.

#### 3.4.1 Are Gabonese populations the source of all African populations sampled?

First, regarding the Gabonese population GA2, the results from the ABC‐RF analyses are most often not conclusive except for the analysis including the population from Uganda (UGA) (Table 2, Appendix S10c): the 10 replicates agreed and favored the hypothesis of both populations originating from a single event of introduction into Africa, UGA being the source of GA2. Apart from this analysis with the population from Uganda (UGA), in the other analyses including the populations from the different African countries, we could most often reject the scenarios of multiple introductions from the same Asian source (MI scenarios) and the ones of a single introduction from the South‐East Asia followed by independent introductions in Africa (SII), which appeared less likely relatively to the scenarios of successive introductions (SIS12 or SIS21; Appendix S10c). Regarding the relationship of GA2 with the population from Nigeria (NGA), all ten replicates agreed with the hypothesis of the Gabonese population GA2 being the source of the Nigerian population (NGA), with nine of them favoring the absence of migration between the two populations after their establishment (Table 2, Appendix S11c). The model choice analysis with GA2 and CA5 as African populations did not allow us to discriminate between the tested scenarios but the low genetic differentiation observed between the two populations (pairwise *F*_ST_ ≈ 0) could indicate that both populations came from the same invasion front.

Second, regarding the analyses that included the Gabonese population GA1, the scenario describing GA1 as the source of the other African populations often had a low percentage of votes (Table 2, Appendices S10 and S11d), suggesting that this population from Gabon could not have been the origin of all the other African populations considered here. In particular, it is excluded that GA1 may have founded GA2 (percentages of votes 2.23%–6.3%), COG (2.37%–6.3%), CA5 (1.87%–5.9%), UGA (0.93%–2.6%), and to a lesser extent CA6 (3.27%–9.73%), and CIV (4.9%–15%). Congruent with this, the population GA1 from Gabon was the African population with the lowest level of gene diversity *H*_s_ and no private alleles.

Regarding the relationship between the two Gabonese populations GA1 and GA2, we could clearly reject the scenario stipulating that GA2 originated from GA1 as well as the hypothesis of recurrent migration between the two populations since their establishment, as all the scenarios that included migration received low support (percentage ranging from 2% to 10.5%, see Table 2, Appendices S10 and S11d). Otherwise, although one replicate disagreed, the analysis of the Gabonese pair of population seemed to favor the hypothesis of successive contamination from a single invasive African source, with GA1 originating from GA2.

Although the low genetic diversity detected in the Gabonese populations might be a consequence from the low sample size of these populations, we still would expect if they were at the origin of the African invasion to detect a relatively high level of diversity in these populations and to observe a relatively low differentiation between these populations and the populations they would have initiated, which is not what we observed here, which supports these results mentioned above.

#### 3.4.2 An Eastern invasion front

Our results highlighted a key role for the population from Uganda (UGA), suggesting that the population from Uganda acted as a bridgehead population for other African populations. The ABC tests including the population of Uganda (UGA) clearly favored the scenario of UGA being the source of the populations NGA from Nigeria, CA5 and CA6 from Cameroon, GA1 from Gabon and CIV from Ivory Coast, without subsequent migration between the source and the newly founded populations. All those tests showed an agreement among the 10 replicates with a relatively high percentage of votes for that scenario relatively to the others and a low to very low support for the scenario of the population from Uganda being the receiving population (Table 2, Appendices S10 and S11e). For the populations from Gabon GA2 and from Congo (COG), this was less clear, as the percentage of votes was relatively lower or in disagreement between the replicated tests. The analyses seemed to nevertheless favor the hypothesis of the population from Uganda (UGA) being the source of the two populations, whether migration between the population UGA and the other two occurred after their establishment or not: without distinguishing between the scenarios with and without migration, the two scenarios with the population UGA as the source of the other (SIS21mig and SI21nomig) received higher support than the other three pairs of scenario, S1S12mig and SIS12nomig, SIImig and SIInomig and MImig and MInomig, as shown by the sum of the percentage of votes (Table 2, Appendices S10 and S11g). Congruently, Uganda had the second highest level of private allelic richness among African populations and showed a relatively low pairwise genetic differentiation from other African populations (Table 1).

#### 3.4.3 Two contrasted patterns of propagation

First, it is worth mentioning that for 8 pairs of populations, the scenarios with recurrent migration after founding consistently found very low support (the percentage of votes was always below 0.15) whatever the historical scenario considered (Table 2, Appendices S10 and S11g). These population pairs concerned four of the seven pairs that include the population from Uganda (pairs UGA and the populations from the Ivory Coast (CIV), the Nigeria (NGA), the population CA5 from Cameroon and GA1 from Gabon) and four of the pairs that include the population GA1 from Gabon with its geographically closest populations (pairs GA1 and the two populations from Cameroon (CA5 and CA6), the other Gabonese population GA2 and the population from Congo (COG)). While the population from Uganda (UGA) appeared to be a source population for the 4 concerned west African populations, the population GA1 from Gabon appeared to be a receiving population. Similarly to the GA1 population, the model choice analysis including the Nigerian population (NGA) most often favored a scenario where it would have been founded by the other African population following a single introduction event from the Asiatic source population and without migration between the African populations after their founding (Table 2, Appendices S10 and S11f). The two exceptions concerned the analysis of the pair with the CIV population from Ivory Coast, which seemed to favor a scenario with migration, and the analysis of the pair with the GA1 population, which favored the scenario of GA1 being founded by NGA. Apart from these two, we could most often clearly reject the scenarios of the Nigerian population (NGA) as a source population for the other African population while identifying the scenario of the Nigerian population as a receiving population as the most likely from our analyses.

These results suggest that those west African populations may have been founded through rare long‐distance dissemination events rather than in the form of a continuous invasion front.

Conversely, a scenario with migration between the African populations considered in the analysis was favored for the population group including the populations from Cameroon, CA5 and CA6, and the population GA2 from Gabon, as well as the populations from Ivory Coast (CIV) and from Congo (COG) although these last two did not exchange individuals with all of the other populations. For these pairs of populations, ABC scenario choice was inconclusive considering the historical scenario of founding (any of the two populations could have founded the other one), but scenarios with migration received more votes than scenarios without migration. The difficulty for those 3‐population tests to discriminate between the different scenarios might come from the presence of recurrent migrations between the two African populations since their establishment. Such a pattern is compatible with the assumption of a continuous invasion front from Gabon to Cameroon and Ivory Coast as described historically.

The population from Congo (COG), which could have been founded by the population from Uganda UGA, might also have played an important role in the establishment of other west African populations, such as the populations from Ivory Coast (CIV), from Nigeria (NGA) or from Gabon (GA1), as it could have served as a bridgehead population through stochastic founder events. Besides, scenarios including migration between the (COG) population from Congo and other west African populations from Cameroon (CA5 and CA6) and from Gabon (GA2) often had higher percentages of votes than the scenarios without migration, indicating recurrent migration between the population COG and the two Cameroonian populations and the Gabonese population GA2. The COG population also presented the highest level of private allelic richness *A*_*r*_ and a relatively high level of gene diversity suggesting older occurrence than the others populations.

## 4 DISCUSSION

### 4.1 The complex history of propagation of *Pseudocercospora fijiensis* in Africa

According to the most commonly expressed historical hypothesis, the invasion of West Africa resulted from the introduction into Gabon of infected plant material brought from Asia, in 1978 (Blomme et al., 2013). Dates of first reports were highly suggestive of a regular front extending from Gabon to Ivory Coast (Mourichon & Fullerton, 1990). Some authors also mentioned the possibility of an introduction of BLSD in East Africa (Pasberg‐Gauhl et al., 2000). Using a systematic comparison of generic scenarios on all pairs of available African populations with ABC‐RF, we revealed a more complex history of propagation of *P. fijiensis* in Africa. We did find support for the existence of a front stemming from Gabon and going through Cameroon to Ivory Coast. Indeed, both between Gabon and Cameroon and between Cameroon and Ivory Coast scenarios including recurrent migration better represented the data than scenarios assuming pure divergence without migration. This suggests that populations from these countries were connected by recurrent gene flow in a stepping‐stone manner, which is consistent with the idea of a continuous front.

Surprisingly though, two samples from Gabon and Cameroon (GA1 and CA6), as well as two samples from neighboring countries (COG from Congo and NGA from Nigeria) were shown to originate from the population from Uganda (in Eastern Africa) rather than from the Western continuous propagation front. Between all five populations, scenarios without migration were preferred over scenarios assuming recurrent gene flow, suggesting a stochastic propagation associated with rare long‐distance migration events between these populations of distant countries.

A recent study of BLSD invasion in an island context (Caribbean archipelago) has demonstrated that the two northern and southern fronts are mainly explained by both historical data and population genetic structure (Carlier et al., 2021). In contrast, in our study, it was impossible to decipher the historical relationships between the two fronts but scenarios where multiple introductions would lead to the formation of the two fronts always received little support, with very low numbers of votes. Thus, we suggest here that all studied populations stemmed from a single introduction onto the continent. However, the present data do not allow to further characterize the origin of this initial introduction event, and to decipher between a scenario where a Gabonese introduction expanded to the West and East Africa, or a scenario where an Eastern introduction found all populations, or a scenario where a more central continental introduction, consistent with an unofficial mention of the disease in Zambia in 1973 (Blomme et al., 2013), independently founded the two fronts. These dissemination routes are in accordance with the low invasion speed (80 km/year) calculated following various approaches (analyses of historical data in Carlier et al., 2021 or a modeling study in Hamelin et al., 2016).

### 4.2 Demographic aspects

Such a pattern of spread seems more complex than expected from the historical knowledge and calls into question the hypothesis of a wind‐dispersed pathogen naturally spreading over a landscape with a continuous distribution of susceptible hosts (large banana production areas surrounded by a diffuse matrix of plantain; see Brown et al., 2002). These findings are strikingly concordant with the landscape genetic studies conducted at a different geographical context (an island context, Carlier et al., 2021) or at finer scales in Africa (tens of kilometers, Rieux et al., 2011), which suggested that *P. fijiensis* might combine both a gradual spread via a regular disease front and more stochastic processes potentially based on long‐distance dispersal events (see also Carlier et al., 2021; Rieux et al., 2014). Such stratified dispersal processes have often been shown to drive biological invasions (e.g., Petit, 2011). For intermediate levels of long‐distance dispersal, stratified dispersal can lead to patchily structured populations (Bialozyt et al., 2006), consistent with the observations presented here. In *P. fijiensis*, stratified dispersal may involve a combination of different processes: natural dispersal, including that mediated by asexual spores over short distances (a few meters) and airborne sexual spores maintained during invasion as suggested in Carlier et al. (2021) and carried over longer distances (from several meters to kilometers), and anthropogenic transport of infected leaves. More exhaustive sampling and further landscape genetics studies would increase our understanding of the contributions of progression via the gradual movement of a front or by stochastic jumps, but the combination of these different natural and anthropogenic mechanisms will remain very difficult to unravel.

### 4.3 The benefits and limits of reconstructing routes of propagation with ABC‐RF

As microscopic organisms in general, plant pathogens may remain unknown or remain undetected for long periods before they produce damaging epidemics on crops. As a result, plant pathologists often lack knowledge of their evolutionary origin and history (Stukenbrock & McDonald, 2008). Reconstructions of the origin or propagation pathways of emerging plant pathogens have already benefited considerably from phylogeographical and population genetic studies in addition to epidemiological data. Genetic approaches have for instance enabled rejecting the hypothesis that *Rhynchosporium secalis* had co‐evolved with its host in the Middle East (Linde et al., 2009). Similarly, the center of origin of the potato late blight pathogen, *Phytophthora infestans*, has been the subject of much debate: descriptive genetic analyses initially suggested a Mexican origin, but coalescent methods subsequently revealed that it emerged in the Andes, following potato domestication (Gómez‐Alpizar et al., 2007). Here as well, a combination of standard population genetics methods and Bayesian inference of population history provide unexpected insight into the invasion of Africa by *P. fijiensis*, completing widely held views derived from field observations.

Model choice analyses using an ABC approach have now been widely used to reconstruct the history of biological invasions. Most often, such analyses result in accepting the scenario with the highest posterior probability, or number of votes, as the most likely scenario out of the tested ones. However, in some cases, there is no clear cut‐off among the number of votes between some of the scenarios so that it is impossible to accept one particular scenario. Yet, in our case, while some scenarios, obtaining similar numbers of votes in the Random Forest procedure, could not be distinguished, others had a number of votes close to 0. Those latter scenarios could therefore be unambiguously rejected, which shed light on the history of *P. fijiensis* in Africa. Although accepting one global scenario that include all populations as the most likely is obviously the objective of such approaches, we showed in this study that rejecting some scenarios can also be fruitful and add some useful information, especially when positive results are not that clear. Similarly, even if it is not possible to reconstruct one unambiguous scenario that would include all the analyzed populations, interpreting partial analyses can add valuable information, for example by allowing one to refute or precise hypotheses on the diffusion of organisms, or highlighting preferential relationships between certain populations. The ambiguity in the results of the ABC analyses could come from the fact that some scenarios may be too similar. The complex demographic dynamics of some organisms such as pathogens, which undergo frequent bottlenecks followed by exponential demographic expansions, or with subtle genetic differentiation and/or recurrent migration between populations may also contribute to ambiguous results in such model choice analyses. As a possible solution, a recent development of the ABC‐RF approach allows to compare groups of scenarios that model similar types of evolutionary events, e.g. scenarios with or without migration (Chapuis et al., 2020; Estoup et al., 2018). However, in our case, the implementation of this approach did not appear to be advantageous compared to the analyses including all the scenarios separately (results not shown).

### 4.4 General conclusion

In conclusion, we show that the colonization of the African continent by the fungal plant pathogen, *P. fijiensis*, has most likely involved a single introduction event into Africa, and both a continuous front of invasion and some long‐distance dispersal events allowing the invasion of the pathogen at larger scales. This study constitutes an additional example that bottlenecks may not necessarily be detrimental to invasion success, contrary to what is widely believed (Frankham, 2005). Here, banana plantations may be considered to be a particularly homogeneous susceptible environment worldwide, as the majority of cultivars (dessert bananas, plantains, East African highland bananas) are very susceptible against the disease with few control methods and are exclusively cultivated in tropical climatic conditions. Hence, *P. fijiensis* had probably adapted to this susceptible environment through contact with similar commercial plantations of cultivars (e.g. Cavendish group) in its area of origin, before its introduction into Africa. The adaptive challenge faced in Africa was therefore probably mild enough for the invasion to succeed regardless of the degree of impoverishment of evolutionary potential during bottlenecks. Scenarios describing previous adaptations of invading populations to widespread human‐altered environments with homogeneous features are increasingly being identified in studies of damaging invasive species (Hufbauer et al., 2012). Such scenarios may apply particularly to crop pests, as modern agricultural practices provide these organisms with the opportunity to adapt to globally standard high‐yield plantations, before propagation by long‐distance exchanges (Guillemaud et al., 2011). Additional examples of demographic inferences in successful emerging fungal plant pathogens should be studied to assess the generality of these ideas.

## AUTHOR CONTRIBUTIONS

**A. Gilabert:** Formal analysis (equal); methodology (equal); writing – original draft (equal). **A. Rieux:** Formal analysis (equal); methodology (equal); writing – review and editing (equal). **S. Robert:** Conceptualization (equal); data curation (equal); formal analysis (equal); methodology (equal); writing – original draft (equal). **R. Vitalis:** Formal analysis (equal); methodology (equal); writing – review and editing (equal). **M.‐F. Zapater:** Data curation (equal); resources (equal). **C. Abadie:** Data curation (equal); resources (equal); writing – review and editing (equal). **J. Carlier:** Data curation (equal); resources (equal); writing – review and editing (equal). **V. Ravigné:** Conceptualization (equal); formal analysis (equal); methodology (equal); supervision (equal); writing – original draft (equal).

## FUNDING INFORMATION

This work was supported by the Région Languedoc‐Roussillon, the Agence Nationale de la Recherche (project EMERFUNDIS ANR 07‐BDIV‐003 and EMILE ANR 09‐BLAN‐0145‐01), and French Agropolis Fondation (Labex Agro – Montpellier, BIOFIS project number 1001‐001 and Labex Agro –Montpellier, E‐SPACE project number 1504‐004). VR was funded by the European Union (European Regional Development Fund, ERDF contract GURDT I2016‐1731‐0006632), the Conseil Régional de La Réunion, and the Centre de Coopération Internationale en Recherche Agronomique pour le Développement (CIRAD).

## Supporting information


Appendix S1

Appendix S2

Appendix S3

Appendix S4

Appendix S5

Appendix S6

Appendix S7

Appendix S8

Appendix S9

Appendix S10

Appendix S11

## Data Availability

The microsatellite dataset, template files for fastsimcoal analyses and R scripts used to generate the tables and figures of the manuscript are deposited in the dryad repository: doi:10.5061/dryad.rn8pk0pgw.
