# Supplementary material for: Revisiting the historical scenario of a disease dissemination using genetic data and Approximate Bayesian Computation methodology: The case of Pseudocercospora fijiensis invasion in Africa
Source: Ecol Evol. 2023 Apr 19;13(4):e10013. doi: 10.1002/ece3.10013 (PMC10116021; doi:10.1002/ece3.10013)
Supplement: Supplementary file 1 — Appendix S1 [file ECE3-13-e10013-s007.docx]

**Appendix A1** - Prior distributions of the model parameters and complex parameters estimated during the ABC-RF analyses.

A) Prior sets for the historical, demographic and mutation parameters used for ABC-RF analyses

|  |  |  |  | Principal prior set | |
| --- | --- | --- | --- | --- | --- |
| Simple parameters | | |  | Distribution | Extreme values |
|  | Effective size of established populations | | |  |  |
|  |  | SEA population | NSEA | Uniform | [1000; 100,000] |
|  |  | Unsampled Asian population | Nsea | Uniform | [1000; 100,000] |
|  |  | Unsampled African founder population(s) | Naf*i* | Uniform | [1000; 100,000] |
|  |  | Sampled African populations | NAF*i* | Uniform | [1000; 100,000] |
|  | Founding effective population sizes | | |  |  |
|  |  | Unsampled African founder population(s) | NbGaf | Uniform | [1; 500] |
|  |  | Sampled African populations | NbAF*i* | Uniform | [1; 500] |
|  | Bottleneck durations | | |  |  |
|  |  | Unsampled African founder population(s) | dbGaf | Uniform | [0; 50] |
|  |  | Sampled African populations | dbAF*i* | Uniform | [0; 50] |
|  | Duration of established populations (in generations) | | |  |  |
|  |  | Unsampled Asian population | dGsea | Uniform | [300; 100,000] |
|  |  | Unsampled African founder population(s) | dGaf*i* | Uniform | [100; 1,000] |
|  | Migration parameters | | |  |  |
|  |  | Duration of the period with migration | dmig | Uniform | [Gmax*; 330] |
|  |  | Duration of the period without migration | dno_mig | Uniform | [0; 200] |
|  |  | Migration rate | MIG | Uniform | [0.001; 0.1] |
|  | Microsatellite parameters | | |  |  |
|  |  | Microsatellite mutation rates | MutSat*j* | Log-uniform | [2E-6; 2E-4] |
|  |  | Geometric parameter for the GSM^1^ model | Pgsm | Log-uniform | [0.1; 0.4] |

* Gmax: oldest sampling time (in generations backwards)

^1^ GSM: Generalized Stepwise Mutation

B) Definition of the complex parameters estimated in the ABC-RF analyses

| Complex parameters | | |  | Formula |
| --- | --- | --- | --- | --- |
|  | Foundation time (in generations backwards) | | |  |
|  |  | Sampled African populations | TAF*i* | TbAF*i +* dbAF*i* |
|  |  | Unsampled African founder population(s) | TGaf*i* | TbGaf*i +* dbGaf |
|  |  | Unsampled Asian population | TGsea | dGsea + TGaf1 %max% Tgaf2 |
|  | Duration of established populations (in generations) | | |  |
|  |  | Sampled African populations | TbAF*i* | dmig*i +* dno_mig*i* |
|  |  | Unsampled African founder populations | TbGaf*i* | TAF*i* + dGaf*i* |
|  | Bottleneck parameters | | |  |
|  |  | Bottleneck intensity | BotAF*i* | NbAF*i* / NAF*i* |
|  |  |  | BotGaf*i* | NbGaf*i* / NGaf*i* |
|  |  | Bottleneck severity | BotSevAF*i* | dbAF*i* / NbAF*i* |
|  |  |  | BotSevGaf | dbGaf / NbGaf |
