# Supplementary material for: Revisiting the historical scenario of a disease dissemination using genetic data and Approximate Bayesian Computation methodology: The case of Pseudocercospora fijiensis invasion in Africa
Source: Ecol Evol. 2023 Apr 19;13(4):e10013. doi: 10.1002/ece3.10013 (PMC10116021; doi:10.1002/ece3.10013)
Supplement: Supplementary file 2 — Appendix S2 [file ECE3-13-e10013-s001.docx]

**Appendix A2** – Evaluation of the stability of the estimation of the prior error rates according to the number of simulated datasets per scenario and to the number of trees in the random forests.

Estimation of the prior error rates according to the number of trees in the RF with a reference table including 10,000, 20,000, 30,000, 40,000 and 50,000 simulated datasets per scenario for the trio of population Phl, Civ and Ca6.
