# Supplementary material for: Revisiting the historical scenario of a disease dissemination using genetic data and Approximate Bayesian Computation methodology: The case of Pseudocercospora fijiensis invasion in Africa
Source: Ecol Evol. 2023 Apr 19;13(4):e10013. doi: 10.1002/ece3.10013 (PMC10116021; doi:10.1002/ece3.10013)

**Appendix A3** – Genetic differentiation and relationships between the African *P. fijiensis* populations.

a) Pairwise *F_ST_* estimated for all pairs of African *P. fijiensis* populations.

All values were significant in permutation tests, other than the pairwise *F_ST_* between CAM5 and GAB2. Population names are as in Table 1.

|  |  | UGA | CA5 | CA6 | CIV | COG | GA1 | GA2 | NGA |
| --- | --- | --- | --- | --- | --- | --- | --- | --- | --- |
| Africa | CA5 | 0.128 |  |  |  |  |  |  |  |
|  | CA6 | 0.142 | 0.195 |  |  |  |  |  |  |
|  | CIV | 0.240 | 0.205 | 0.276 |  |  |  |  |  |
|  | COG | 0.231 | 0.344 | 0.316 | 0.421 |  |  |  |  |
|  | GA1 | 0.157 | 0.397 | 0.373 | 0.555 | 0.449 |  |  |  |
|  | GAB2 | 0.193 | -0.014 | 0.297 | 0.307 | 0.442 | 0.538 |  |  |
|  | NGA | 0.138 | 0.264 | 0.392 | 0.402 | 0.410 | 0.355 | 0.373 |  |
| SEA | PHL | 0.426 | 0.416 | 0.481 | 0.532 | 0.482 | 0.460 | 0.368 | 0.521 |

b) Unrooted neighbour-joining tree based on Cavalli-Sforza chord distance.

Only two nodes had a percentage of bootstrap support value above 50%. Population names are as in Table 1 and are coloured according to the results of the clustering analysis for K = 5, with populations with mixed ancestries in multiple clusters in grey and population PHL from the Philippines in black (see the section Clustering analyses and Figure 3).


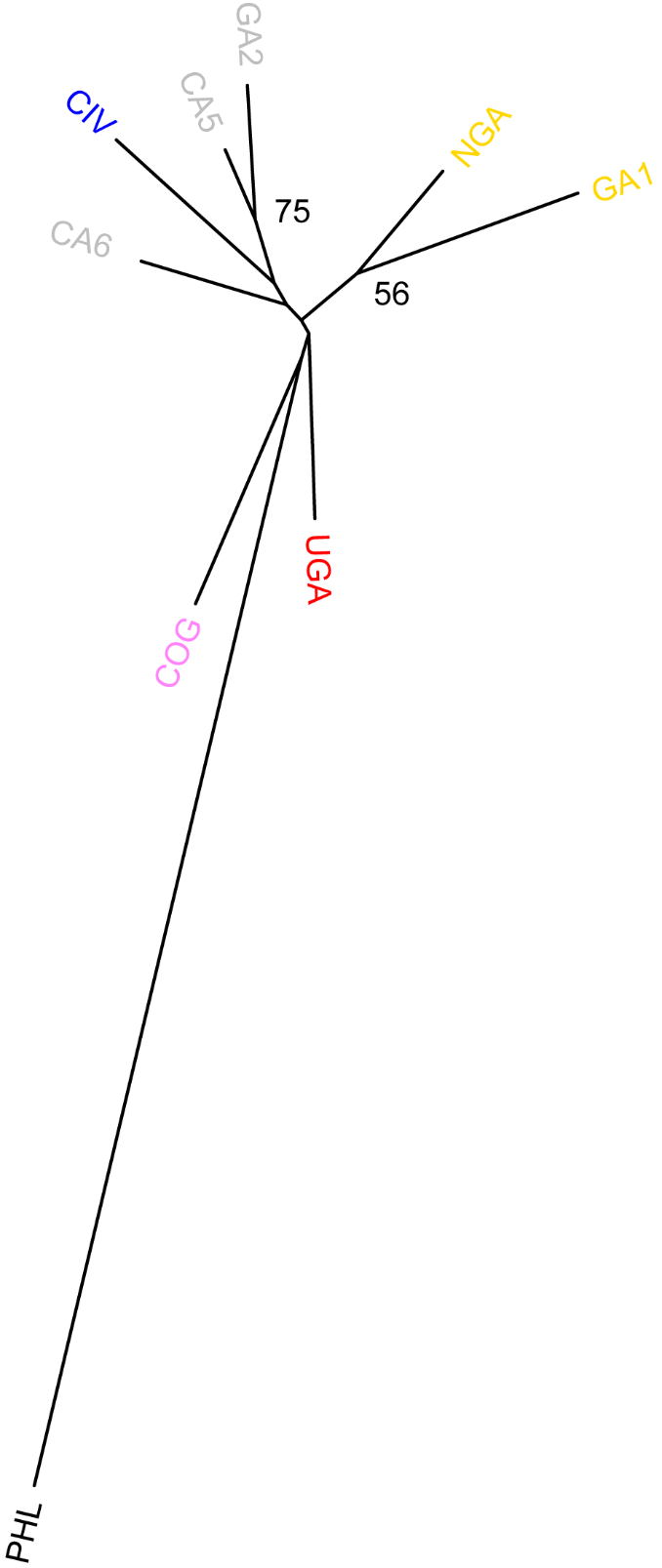

Supplement: Supplementary file 3 — Appendix S3 [file ECE3-13-e10013-s004.docx]
