# Supplementary material for: Revisiting the historical scenario of a disease dissemination using genetic data and Approximate Bayesian Computation methodology: The case of Pseudocercospora fijiensis invasion in Africa
Source: Ecol Evol. 2023 Apr 19;13(4):e10013. doi: 10.1002/ece3.10013 (PMC10116021; doi:10.1002/ece3.10013)

**Appendix A4** - Estimation of the optimal number of clusters *K* in the Structure analysis considering all the African populations.

a) Mean (± SD) log probabilities of the data LnP(D|K) over 10 Structure replicated runs plotted as a function of the putative number of clusters *K*.


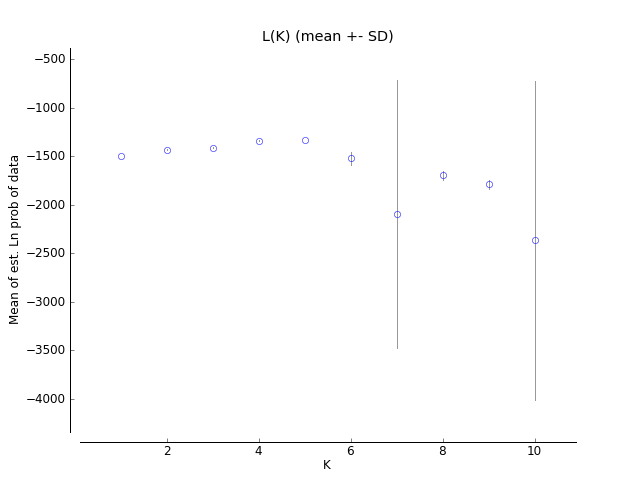


b) Δ*K* plot, following Evanno *et al.* ([2005](https://www.zotero.org/google-docs/?2SJDc7)).


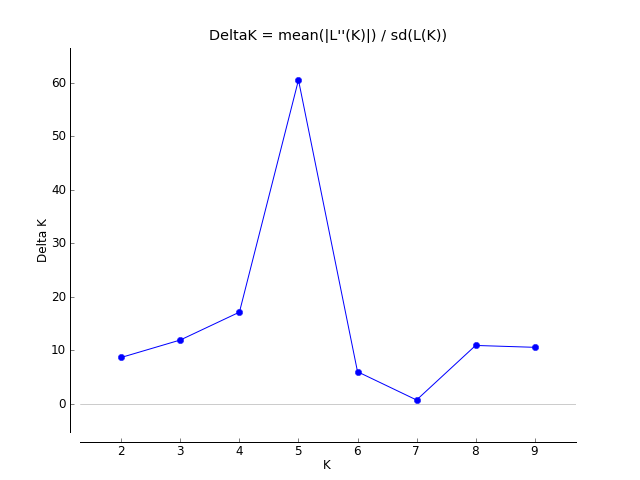

Supplement: Supplementary file 4 — Appendix S4 [file ECE3-13-e10013-s005.docx]
