# Supplementary material for: Revisiting the historical scenario of a disease dissemination using genetic data and Approximate Bayesian Computation methodology: The case of Pseudocercospora fijiensis invasion in Africa
Source: Ecol Evol. 2023 Apr 19;13(4):e10013. doi: 10.1002/ece3.10013 (PMC10116021; doi:10.1002/ece3.10013)
Supplement: Supplementary file 5 — Appendix S5 [file ECE3-13-e10013-s002.docx]

**Appendix A5** - Bayeasian clustering of the African populations without considering the more recently sampled population from Congo, COG population.

a) Mean (± SD) log probabilities of the data LnP(D|K) over 10 Structure replicated runs plotted as a function of the putative number of clusters *K*.


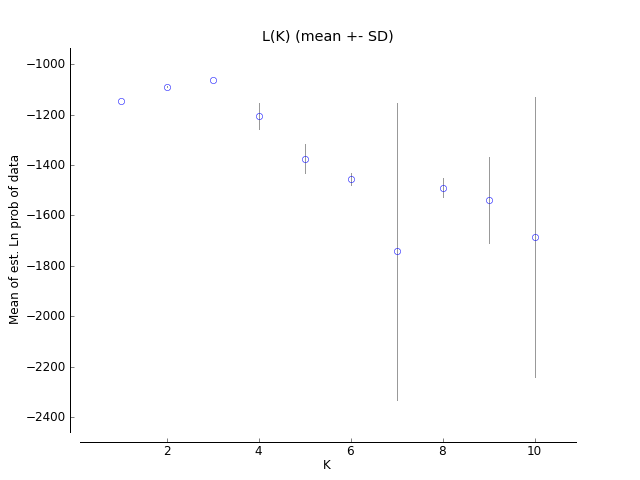


b) Δ*K* plot, following Evanno *et al.* ([2005](https://www.zotero.org/google-docs/?2SJDc7)).


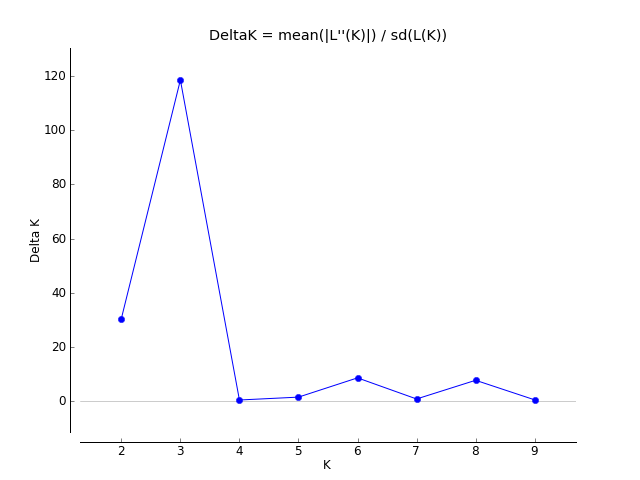


c) Clustering of the multilocus microsatellite haplotypes using STRUCTURE for *K*=2 and *K*=3. Each individual is represented by a vertical line, divided into up to *K*

colored segments representing the individual’s estimated likelihood of membership of each of the *K* clusters. Vertical black lines separate individuals from the different populations of origin, as indicated by population abbreviations under the bar plot (detailed in Table 1).

*K=*2

*K=*3
