# Supplementary figures and images for: Revisiting the historical scenario of a disease dissemination using genetic data and Approximate Bayesian Computation methodology: The case of Pseudocercospora fijiensis invasion in Africa
Source: Ecol Evol. 2023 Apr 19;13(4):e10013. doi: 10.1002/ece3.10013 (PMC10116021; doi:10.1002/ece3.10013)

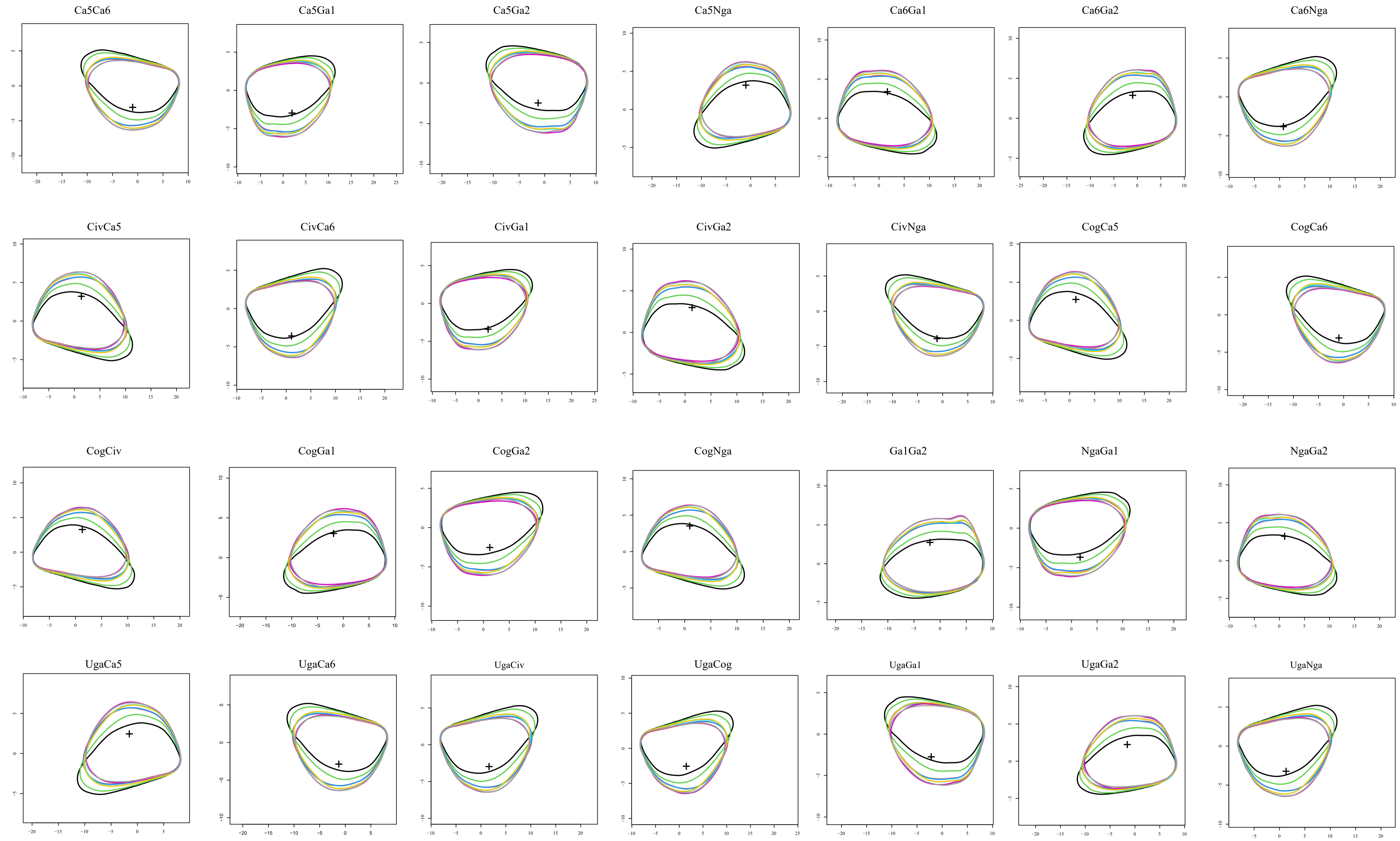

Supplement: Supplementary file 6 — Appendix S6 [file ECE3-13-e10013-s003.pdf]

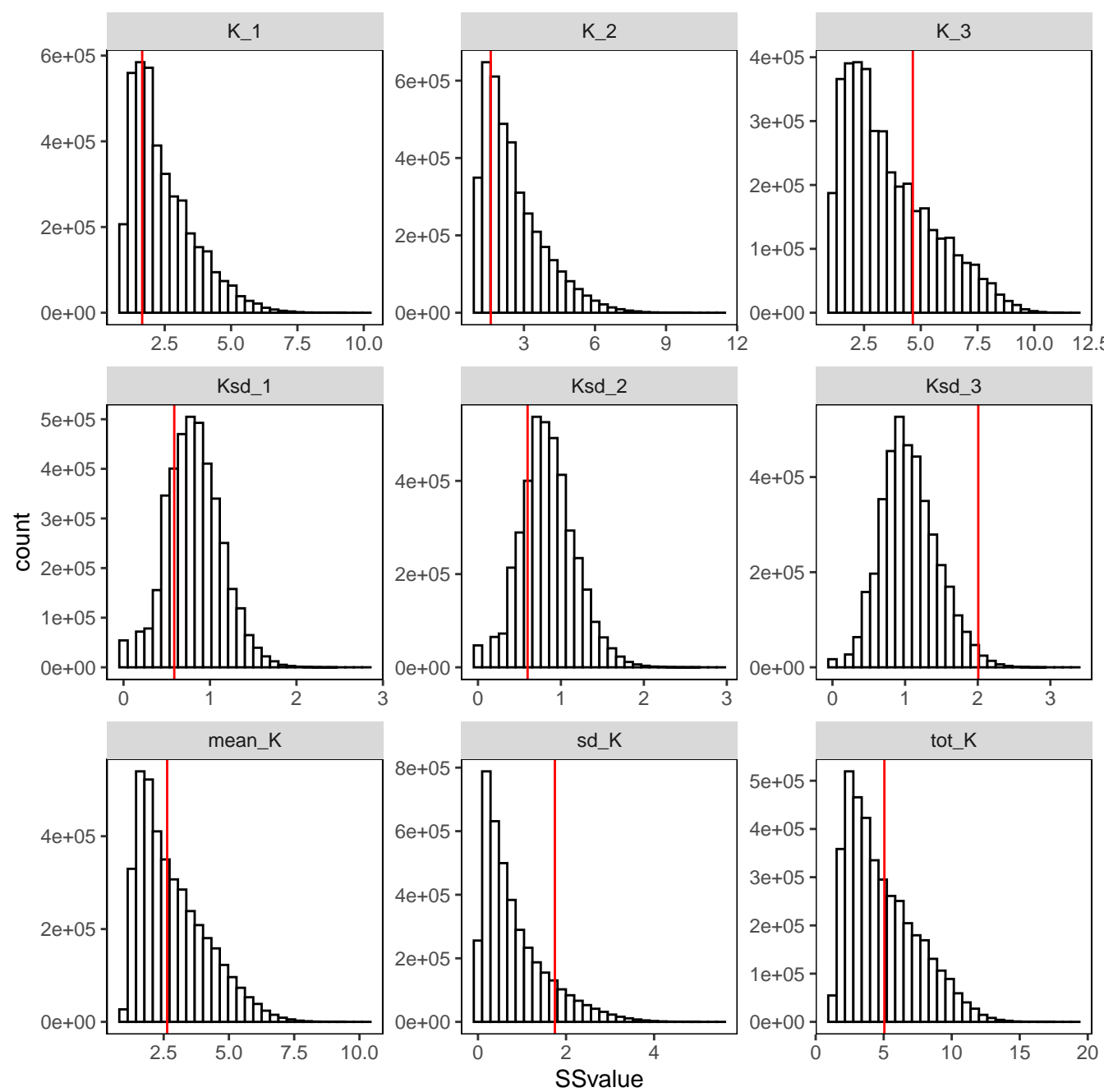

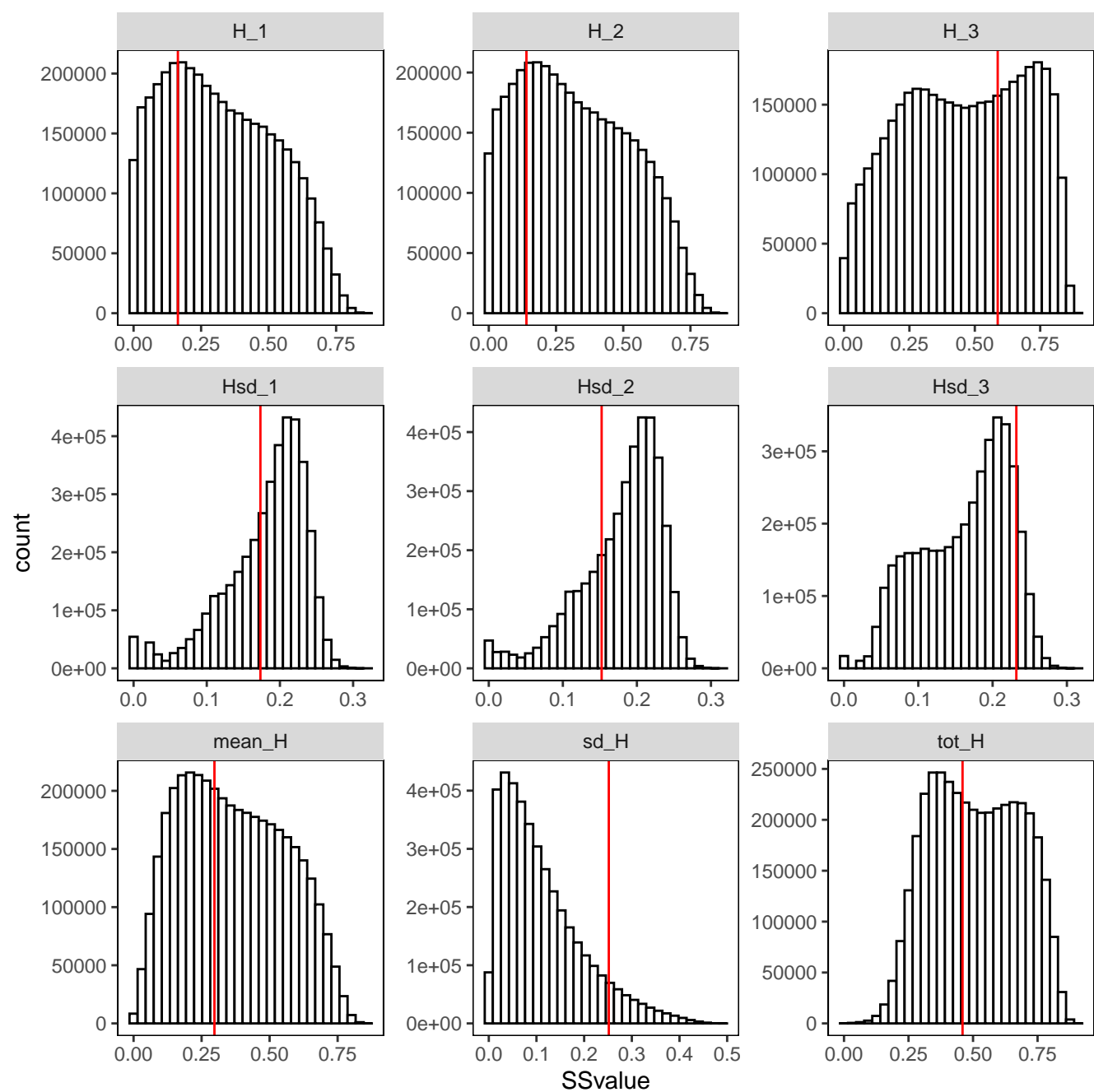

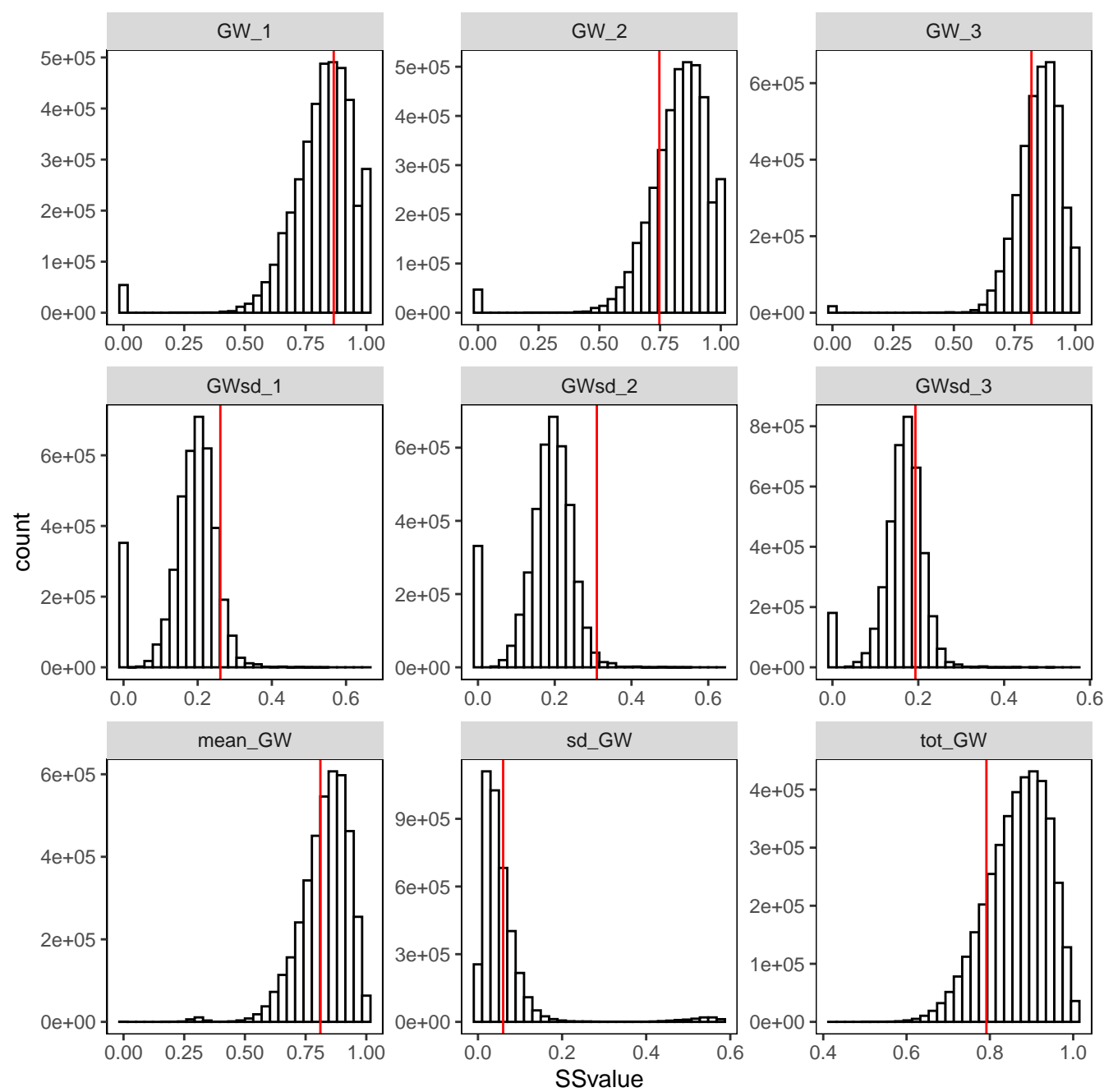

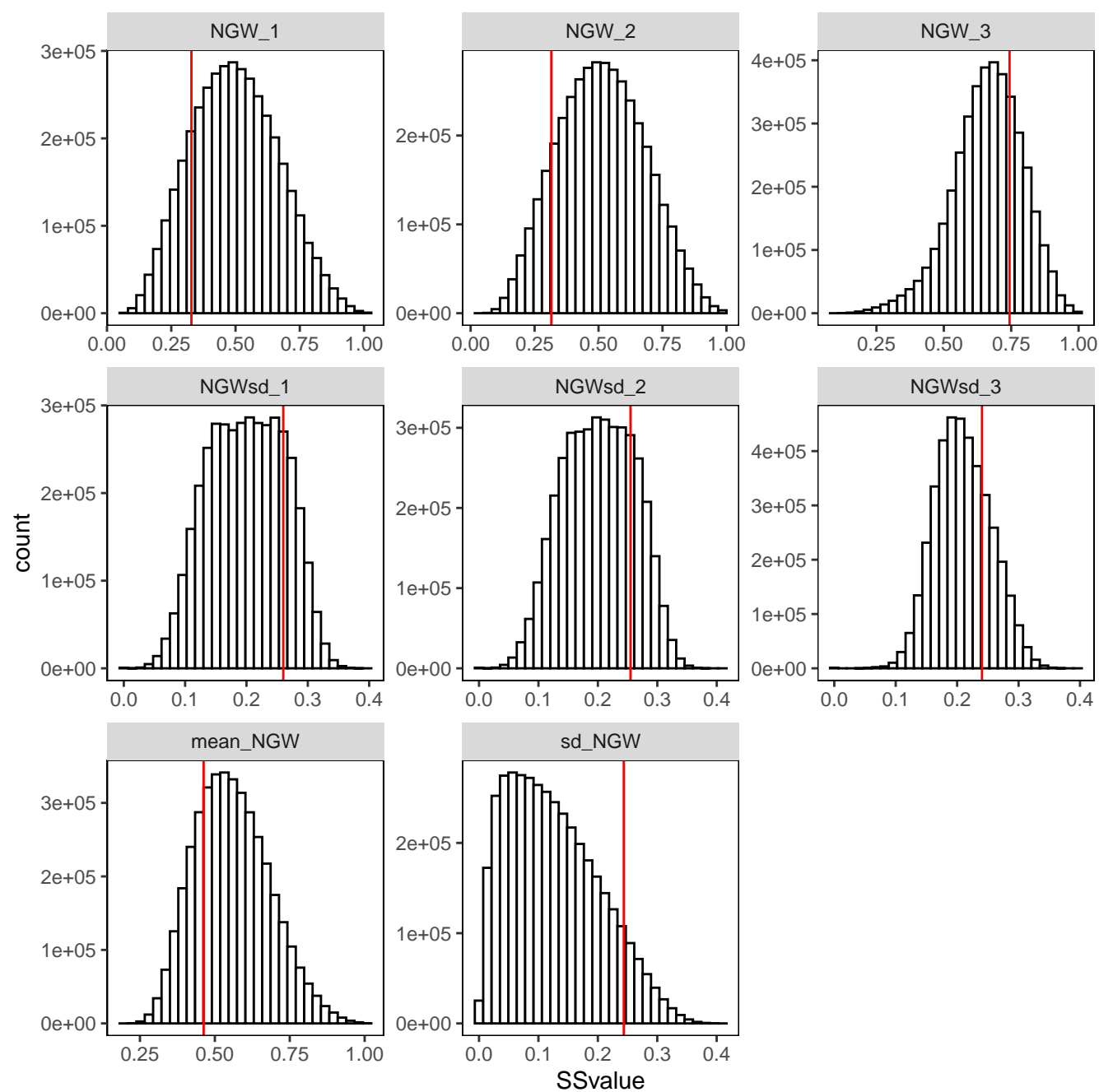

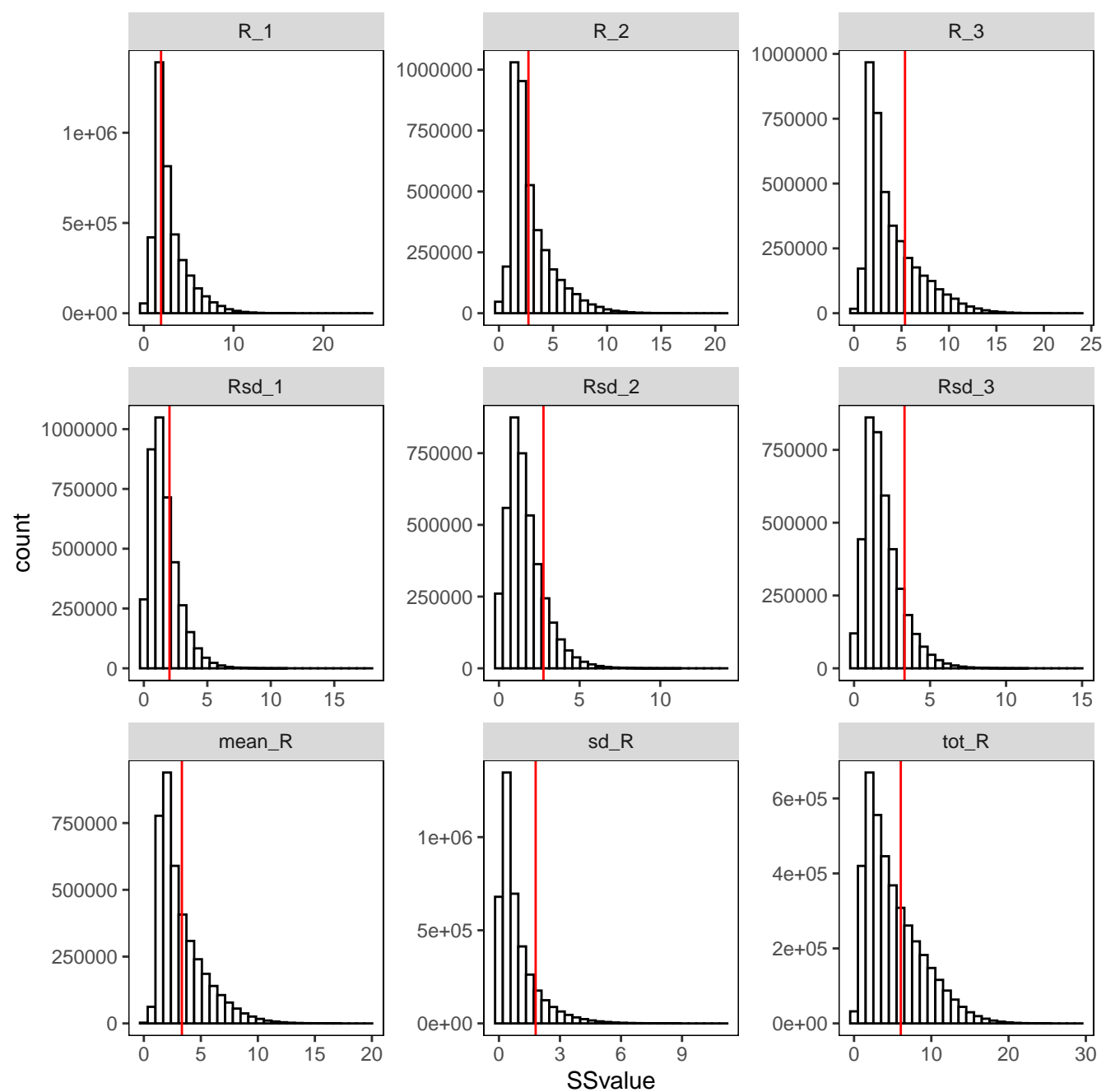

Supplement: Supplementary file 7 — Appendix S7 [file ECE3-13-e10013-s008.pdf]
