# Supplementary material for: Revisiting the historical scenario of a disease dissemination using genetic data and Approximate Bayesian Computation methodology: The case of Pseudocercospora fijiensis invasion in Africa
Source: Ecol Evol. 2023 Apr 19;13(4):e10013. doi: 10.1002/ece3.10013 (PMC10116021; doi:10.1002/ece3.10013)

**Appendix A9** – Variable importance plots for tow population triplets, the triplet PHL-CIV-GA1 (a) and the triplet PHL-COG-CA5 (b). For each population triplet, the variable importance plot for one replicate is given (i), as well as a representation of the ranking of the 8 most important variables according to the replicates (ii).

1. Variable importance plot for the population triplet PHL-CIV-GA1
   1. Variable importance plot for the first replicate

- 1. Ranking of 8 most important variables according to the replicates


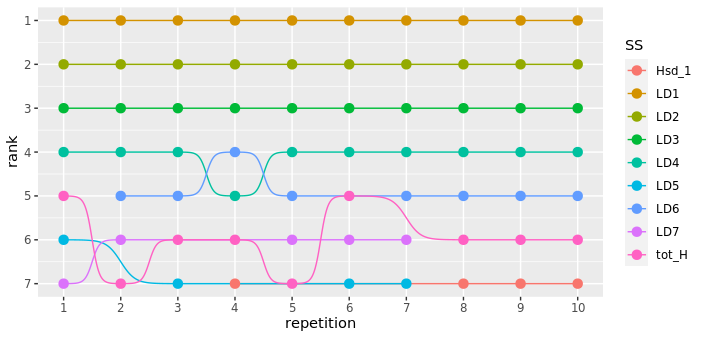


1. Variable importance plot for the population triplet PHL-COG-CA5
   1. Variable importance plot for the first replicate

- 1. Ranking of 8 most important variables according to the replicates


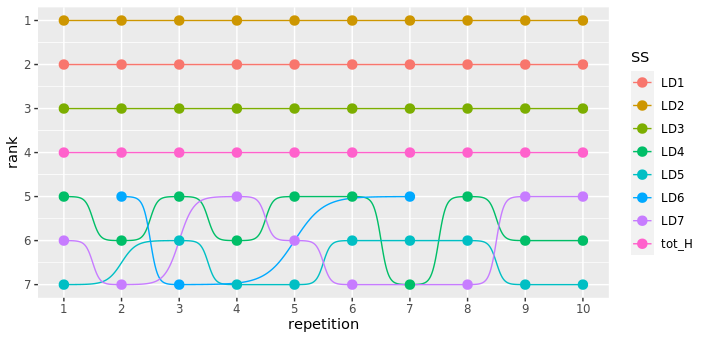

Supplement: Supplementary file 9 — Appendix S9 [file ECE3-13-e10013-s009.docx]
