# Supplementary material for: Revisiting the historical scenario of a disease dissemination using genetic data and Approximate Bayesian Computation methodology: The case of Pseudocercospora fijiensis invasion in Africa
Source: Ecol Evol. 2023 Apr 19;13(4):e10013. doi: 10.1002/ece3.10013 (PMC10116021; doi:10.1002/ece3.10013)

**Appendix A11** – Summary of the results of the model choice analyses

1. Distribution of the percentage of votes per simulated scenario, over all the population triplets analyzed. The red line corresponds to a percentage of vote of 10%.


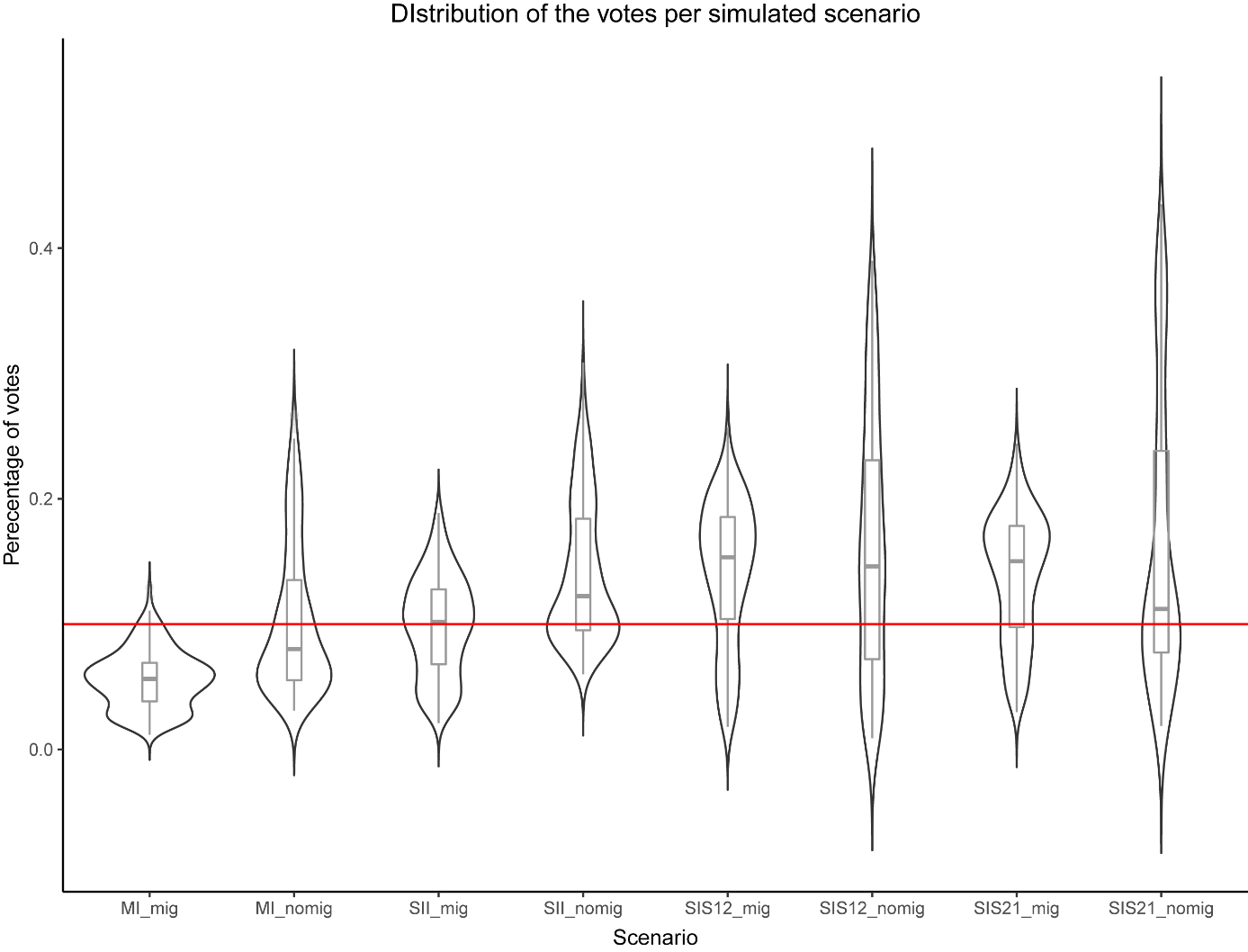


1. Percentage of votes for each scenario and for each 3-population tests, averaged over the ten replicates.


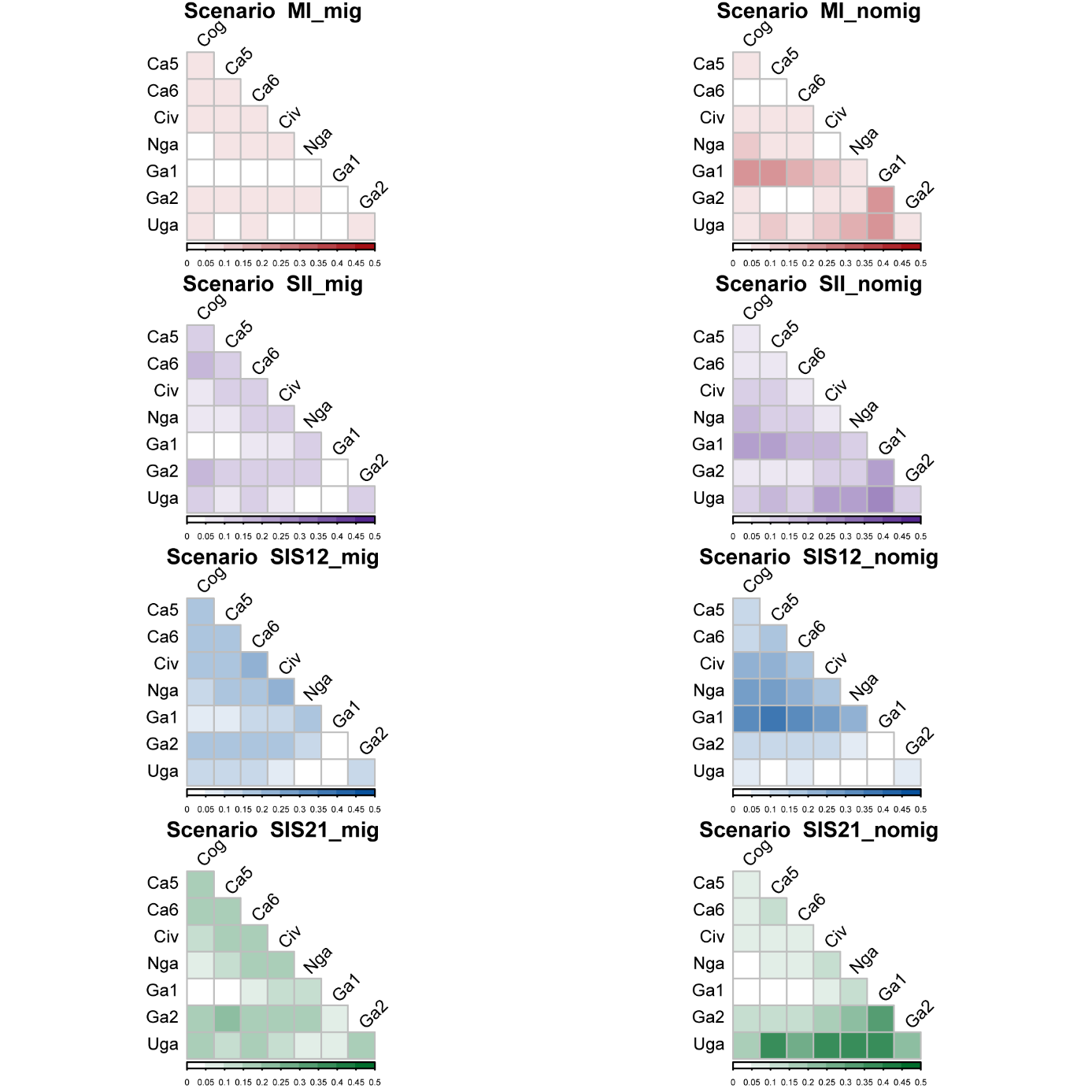


1. Distribution of the percentage of votes per simulated scenario for all 3-population tests that included the population from Gabon GA2, (i) considering the four types of scenario irrespective to migration (MI, SII, SIS12 or SIS21) or (ii) considering separately the eight scenarios tested here.


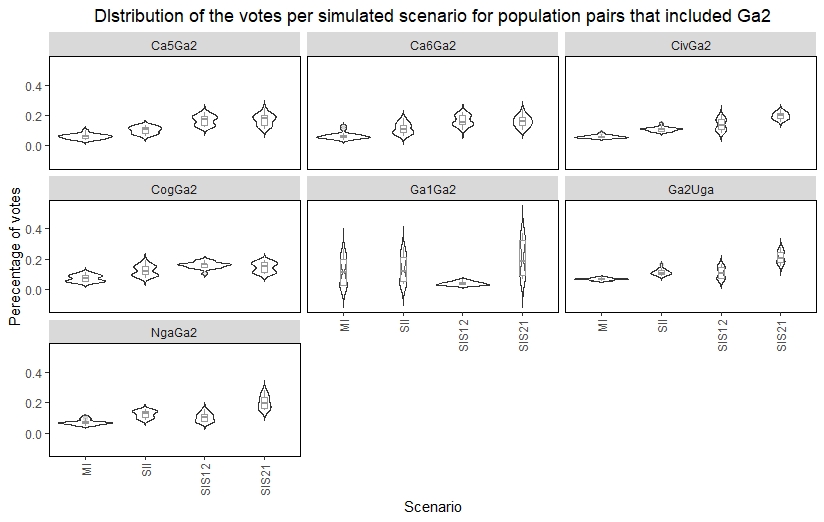


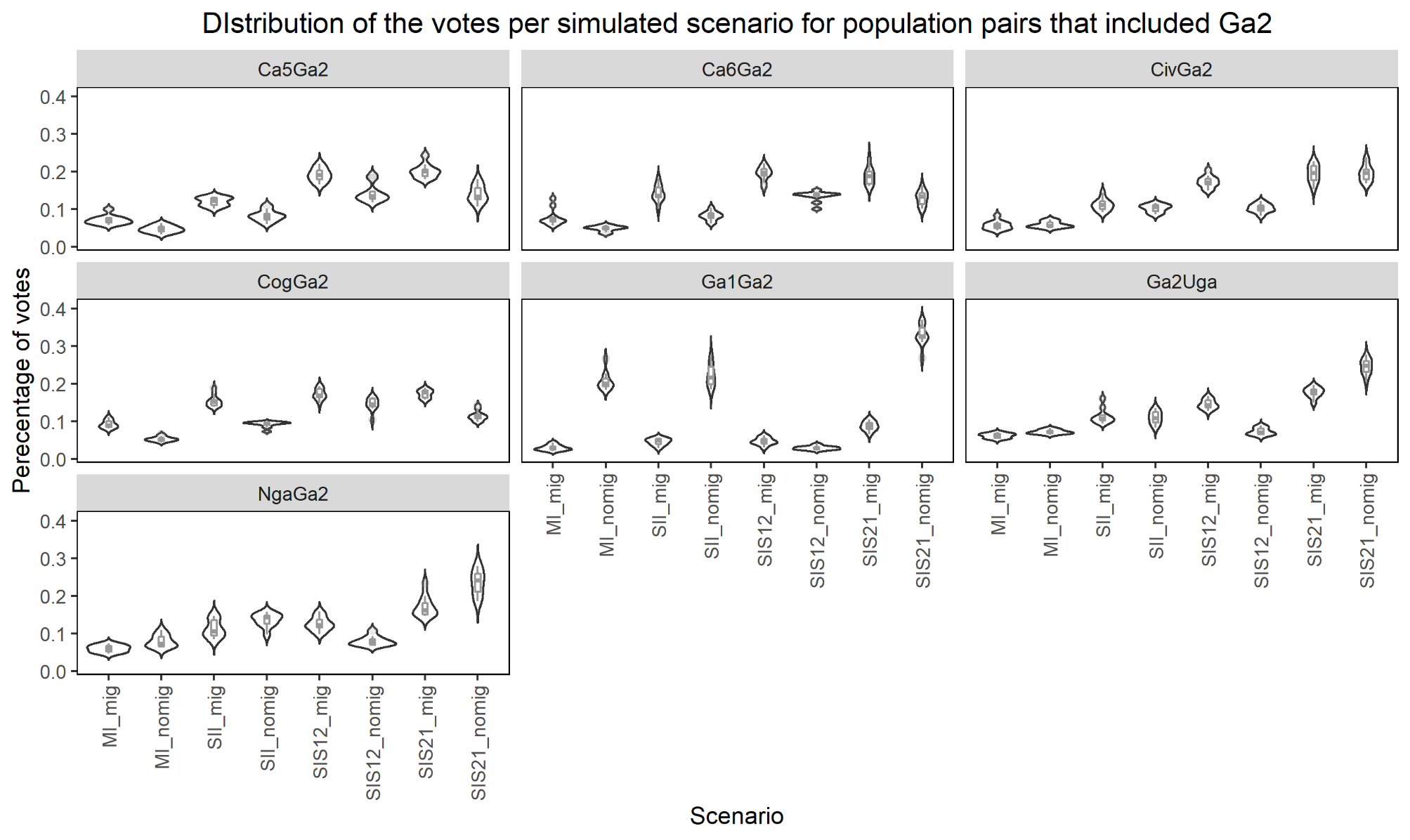


1. Distribution of the percentage of votes per simulated scenario for all 3-population tests that included the population from Gabon GA1, (i) considering the four types of scenario irrespective to migration (MI, SII, SIS12 or SIS21) or (ii) considering separately the eight scenarios tested here.


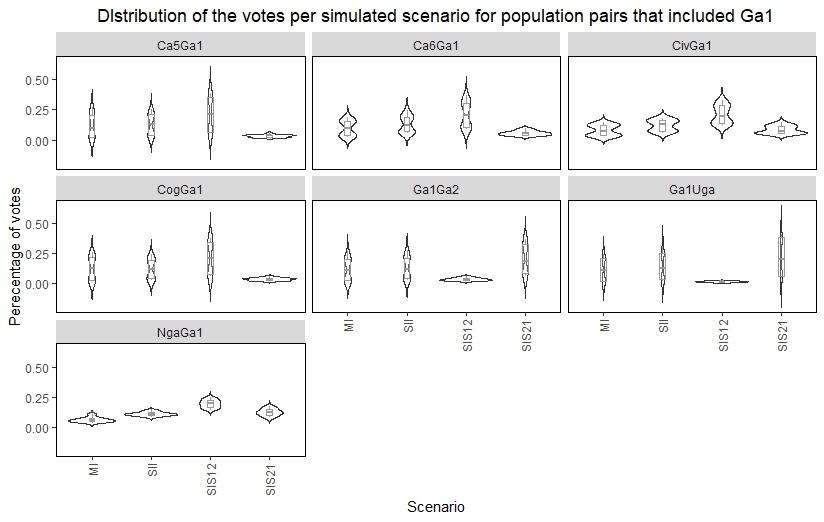


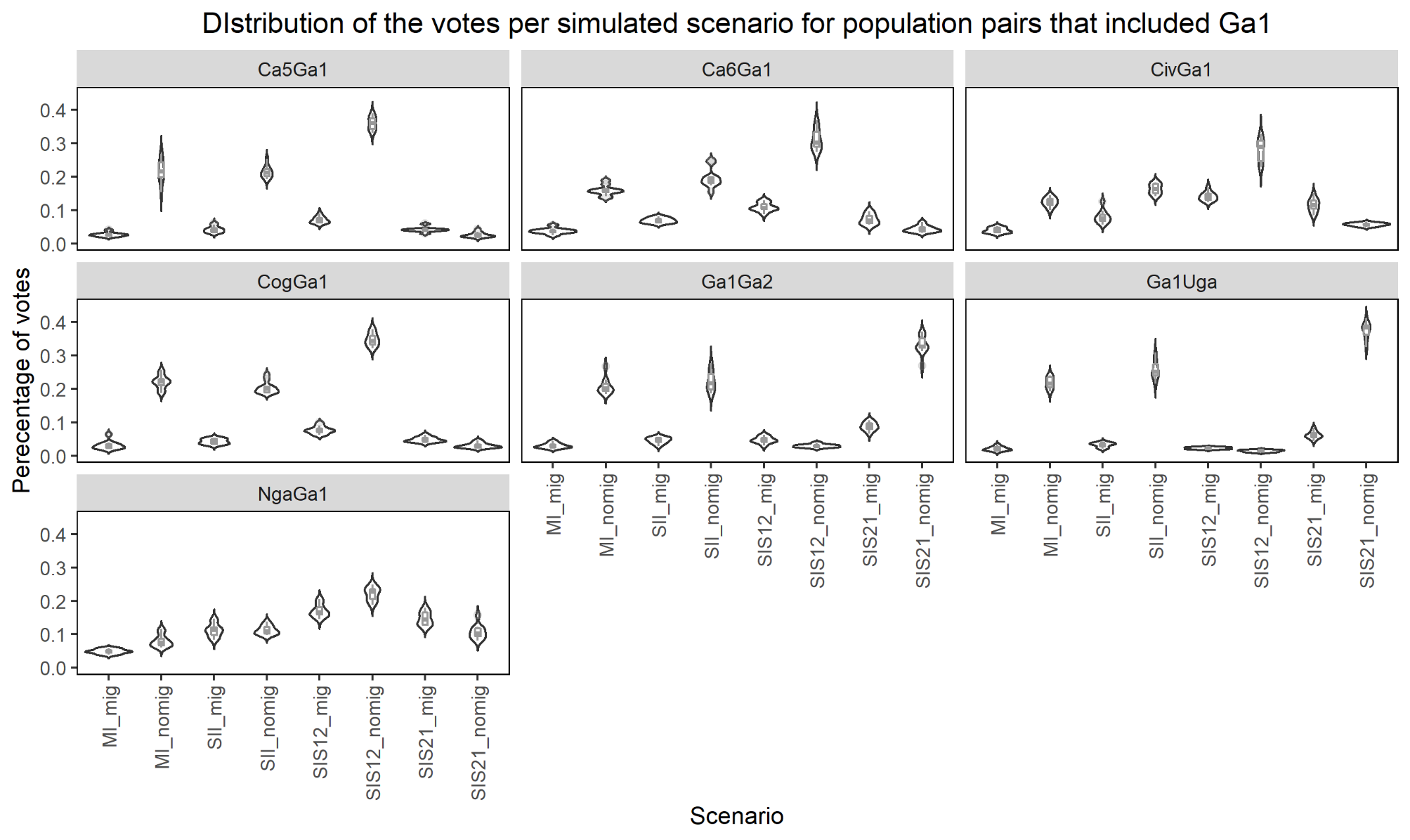


1. Distribution of the percentage of votes per simulated scenario for all 3-population tests that included the population from Uganda (UGA)


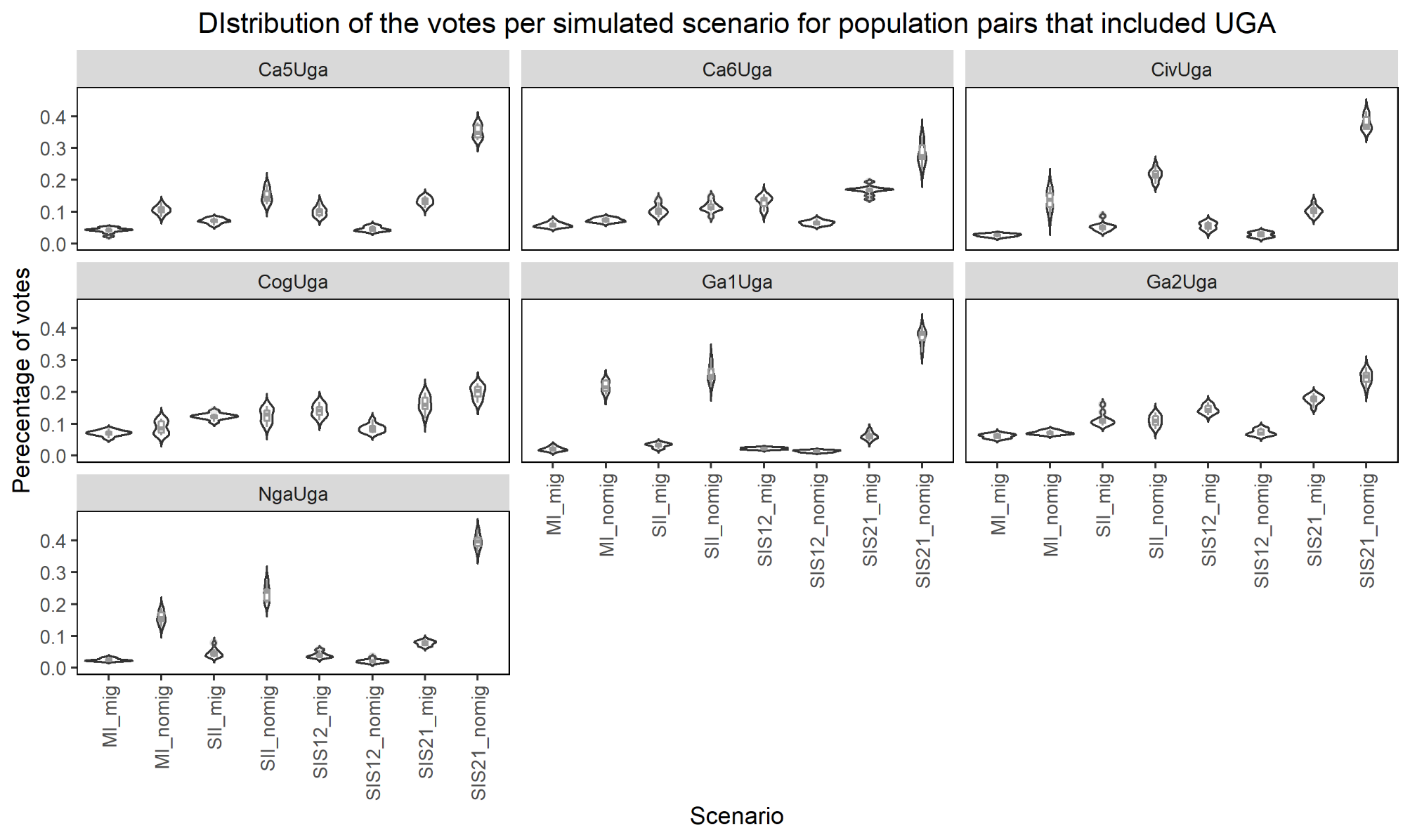


1. Distribution of the percentage of votes per simulated scenario for all 3-population tests that included the populations from Cameroon CA5 and CA6, Ivory Coast CIV, Congo COG and Nigeria NGA.


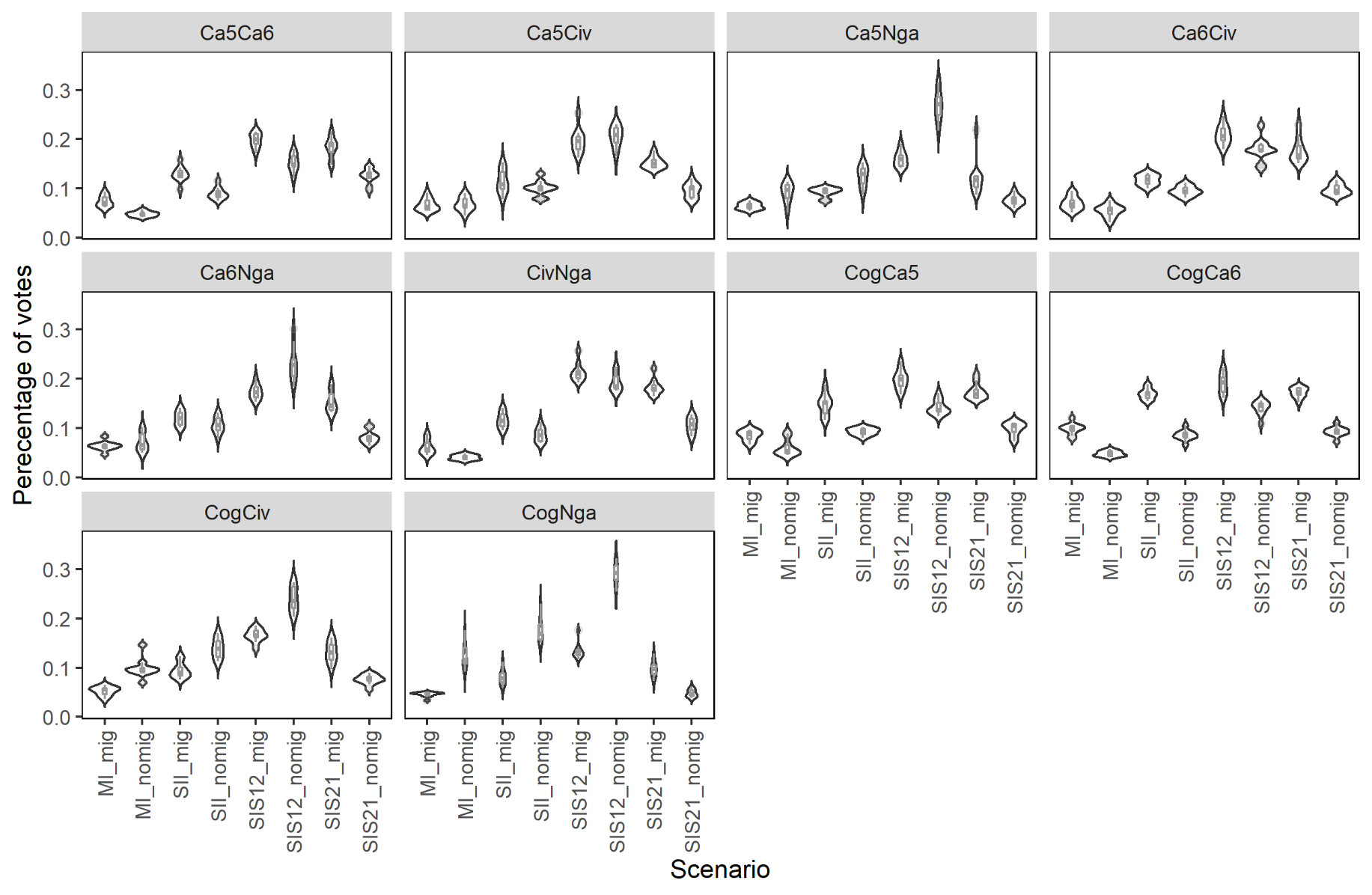


1. Distribution of the percentage of votes for the scenarios with and without migration for all 3-population tests
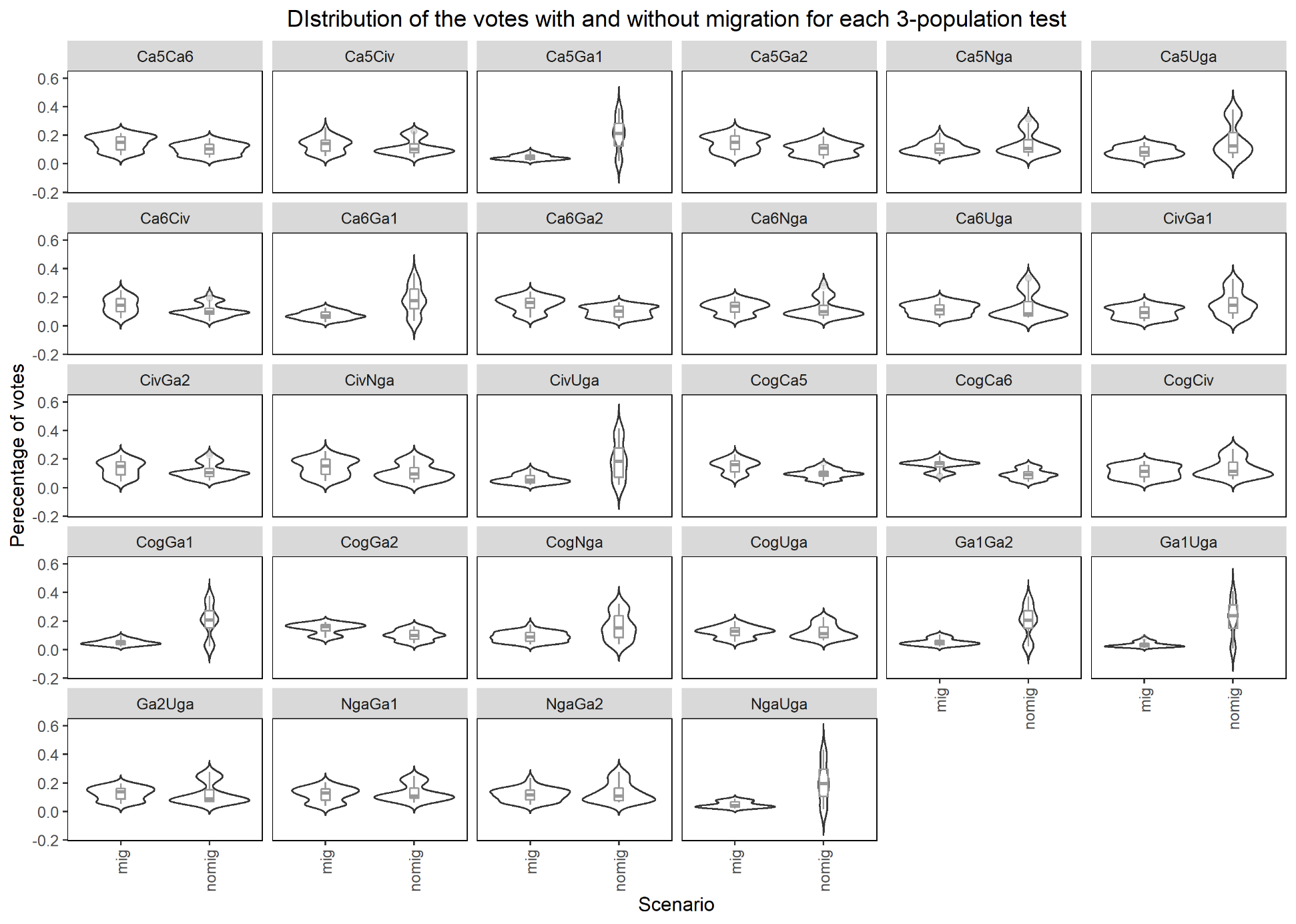

Supplement: Supplementary file 11 — Appendix S11 [file ECE3-13-e10013-s006.docx]
